# Targeting WEE1 enhances the antitumor effect of *KRAS-*mutated non-small cell lung cancer harboring *TP53* mutations

**DOI:** 10.1016/j.xcrm.2024.101578

**Published:** 2024-05-21

**Authors:** Koji Fukuda, Shinji Takeuchi, Sachiko Arai, Shigeki Nanjo, Shigeki Sato, Hiroshi Kotani, Kenji Kita, Akihiro Nishiyama, Hiroyuki Sakaguchi, Koshiro Ohtsubo, Seiji Yano

**Affiliations:** 1 Division of Medical Oncology, Cancer Research Institute, Kanazawa University, Kanazawa, Japan; 2 Nano Life Science Institute, Kanazawa University, Kanazawa, Japan; 3 Department of Respiratory Medicine, Faculty of Medicine, Institute of Medical, Pharmaceutical, and Health Sciences, Kanazawa University, Kanazawa, Japan; 4 Central Research Resource Branch, Cancer Research Institute, Kanazawa University, Kanazawa, Japan

**Keywords:** non-small cell lung cancer, TP53, Kirsten rat sarcoma, KRAS, G12C, WEE1, sotorasib, azenosertib, DNA damage response pathway, CHK2

## Abstract

The clinical development of Kirsten rat sarcoma virus (*KRAS*)-G12C inhibitors for the treatment of *KRAS*-mutant lung cancer is limited by the presence of co-mutations, intrinsic resistance, and the emergence of acquired resistance. Therefore, innovative strategies for enhancing apoptosis in *KRAS*-mutated non-small cell lung cancer (NSCLC) are urgently needed. Through CRISPR-Cas9 knockout screening using a library of 746 crRNAs and drug screening with a custom library of 432 compounds, we discover that WEE1 kinase inhibitors are potent enhancers of apoptosis, particularly in *KRAS*-mutant NSCLC cells harboring *TP53* mutations. Mechanistically, WEE1 inhibition promotes G2/M transition and reduces checkpoint kinase 2 (CHK2) and Rad51 expression in the DNA damage response (DDR) pathway, which is associated with apoptosis and the repair of DNA double-strand breaks, leading to mitotic catastrophe. Notably, the combined inhibition of *KRAS*-G12C and WEE1 consistently suppresses tumor growth. Our results suggest targeting WEE1 as a promising therapeutic strategy for *KRAS*-mutated NSCLC with *TP53* mutations.

## Highlights

• WEE1 inhibitors enhance apoptosis in *KRAS*-mutant NSCLC with *TP53* mutations
• WEE1 inhibition triggers G2/M transition and disrupts CHK2-mediated DNA repair
• Combined KRAS-G12C and WEE1 inhibition suppresses tumor growth in mouse models

## Graphical abstract

**Figure:**
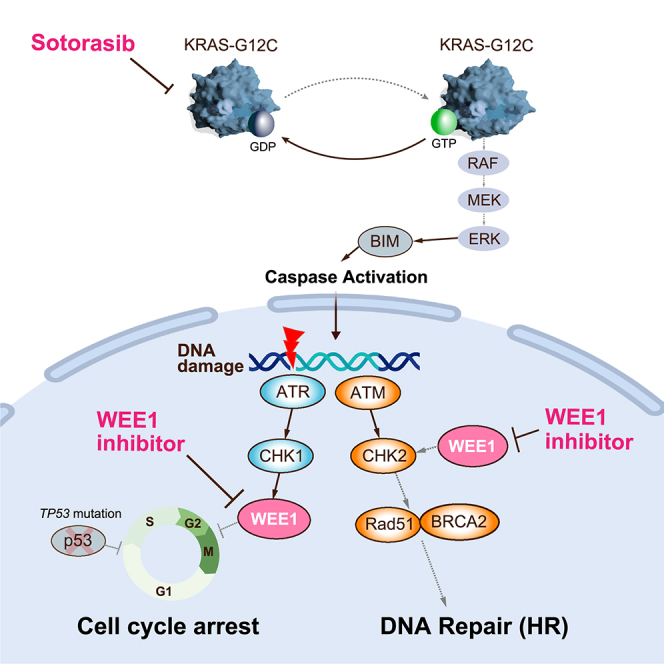

## Teaser

Fukuda et al. show that combining WEE1 kinase and KRAS-G12C inhibitors nearly eradicates *KRAS*-mutant NSCLC with *TP53* mutations in mouse models. This approach disrupts the DNA repair mechanisms of cancer cells, offering an innovative therapeutic strategy for this challenging cancer type.

## Introduction

Kirsten rat sarcoma virus (*KRAS*) mutations are prevalent genetic alterations in cancer and are found in 20%–25% and 10%–15% of adenocarcinoma cases in Western populations and Asia, respectively.^1^^,^^2^^,^^3^ Most *KRAS* mutations occur in codons 12 and 13, with the *KRAS*-G12C mutation being the most common, accounting for 39% of *KRAS*-mutant non-small cell lung cancers (NSCLCs).^2^^,^^3^^,^^4^ Despite the potential for direct inhibition of RAS, its smooth surface structure and low affinity for guanosine triphosphate/guanosine diphosphate have led to the notion that RAS is an undruggable target.^5^

Several small-molecule covalent inhibitors of *KRAS*-G12C have recently been developed, with sotorasib and adagrasib being the most advanced inhibitors currently used in clinical trials. The CodeBreaK 100 phase 2 trial of sotorasib showed promising anticancer activity, with 37.1% of patients yielding an objective response and exhibiting a median response duration of 11.1 months.^6^ Similarly, a KRYSTAL-1 phase 1–2 study of adagrasib demonstrated encouraging results, with 42.9% of patients achieving an objective response and a median response duration of 12.6 months.^7^ These results expedited their approval by the US Food and Drug Administration for the treatment of locally advanced or metastatic NSCLC with *KRAS-*G12C mutations for patients who have received at least one prior systemic therapy.

Despite the encouraging results observed in the treatment of patients with *KRAS*-G12C-mutant NSCLC, both initial and acquired resistance can limit the efficacy of targeted therapies, as observed in *EGFR*-mutant NSCLC and *ALK*-translocated NSCLC.^8^^,^^9^ On-target mutations in oncogenes are a common mechanism underlying acquired resistance to targeted cancer therapies, including tyrosine kinase and mitogen-activated protein kinase pathway inhibitors. Recent clinical trials have revealed multiple acquired *KRAS* alterations in patients with drug resistance, including G12D/R/V/W, G13D, Q61H, R68S, H95D/Q/R, and Y96C/D mutations.^10^ These mutations directly disrupt the binding interaction and resistance to sotorasib and/or adagrasib because the amino acids at positions 12, 68, 95, and 96 are involved in the drug-protein interface.

Moreover, *KRAS*-mutant NSCLC is a highly heterogeneous disease characterized by a high rate of co-mutations, mostly involving *TP53*, *STK11*, and *KEAP1* mutations, which significantly modulate the composition of the tumor microenvironment and consequently affect clinical responses to both immunotherapy and targeted inhibitors currently available in clinical practice.^11^ Recently, different combination strategies, including the inhibition of SHP2, SOS1, and *KRAS-*G12C downstream effectors, as well as the addition of immunotherapy and/or chemotherapy to targeted therapy, have been developed.^12^ Additionally, compared to that of both EGFR and ALK inhibitors, KRAS-G12C inhibitors have shown relatively shorter progression-free survival (PFS),^6^^,^^7^ emphasizing the need for more effective treatment options. This has necessitated the urgent identification of the optimal treatment for patients with *KRAS*-mutated NSCLC.

In this study, to identify potential therapeutic targets that are effective against *KRAS*-mutated NSCLC, we conducted a CRISPR-Cas9 knockout screen using a crRNA library and a drug screen using a custom library of compounds. This approach identified the DNA damage response (DDR) pathway, specifically WEE1 kinase, as having synthetic lethal potential. Although the role of WEE1 in the DDR pathway is well established, our research uncovers an additional aspect of its function in DNA repair mechanisms that are specific to *KRAS*-mutated NSCLC. We also determined that the combined inhibition of *KRAS*-G12C and WEE1 consistently suppressed tumor growth in *KRAS*-G12C-mutated NSCLC with *TP53* mutations. These findings indicate that WEE1 inhibition is crucial in enhancing the efficacy of *KRAS*-G12C-targeted therapies.

## Results

### Target screening using CRISPR-Cas9 library in *KRAS*-mutated lung cancer

**Figure 1:**
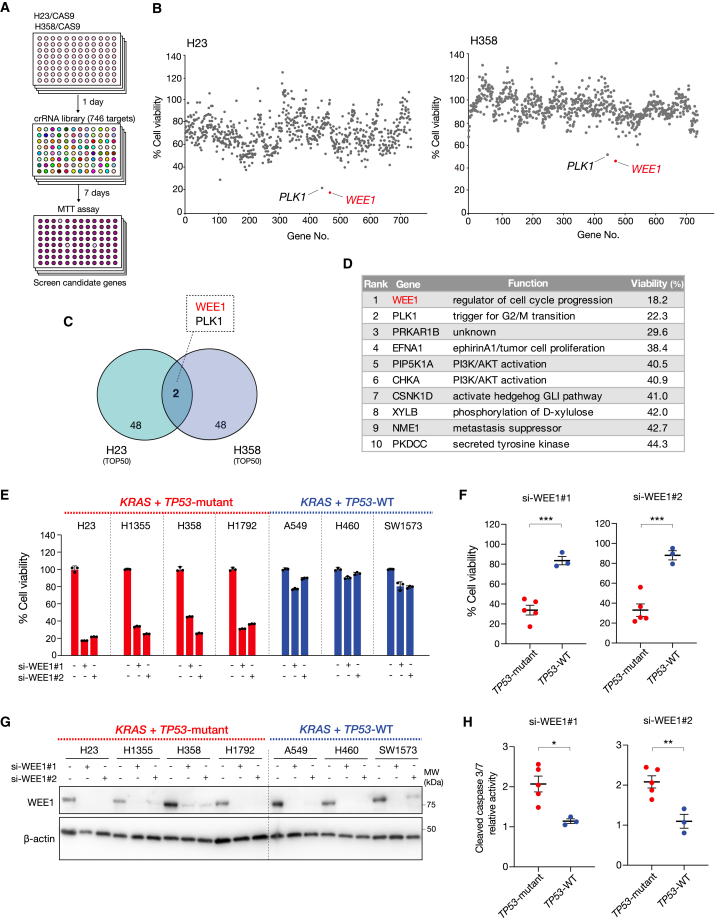
Target screening using CRISPR-Cas9 library in *KRAS*-mutated lung cancer (A) Schematic of functional genomic CRISPR-KO screening. (B) H23 and H358 cells were expressed using Cas9 and treated with a crRNA library for 7 days. Cell viability was assessed using an MTT assay at 72 h. (C) The Venn diagram shows the top 50 genes that suppress growth inhibition of H23 and H358 cells. (D) The top 10 genes that suppress growth inhibition of H23. (E) Cell viability of H23, H1355, H358, H1792, A549, H460, and SW1573 cells transfected with siRNAs targeting WEE1 for 72 h. Cell viability was quantified using an MTT assay. Bars represent mean ± SD of triplicate. (F) Cell viability of *TP53-*mutant and *TP53* wild-type (WT) *KRAS*-mutated lung cancer cells transfected with siRNAs targeting WEE1 for 72 h were compared. Bars represent mean ± SD of triplicate. Statistical significance was determined using Student’s t test. ∗∗∗*p* < 0.001. (G) Cell lysates were analyzed by western blotting with the indicated antibodies. (H) Apoptosis of *TP53-*mutant and *TP53*-WT *KRAS*-mutated lung cancer cells transfected with siRNAs targeting WEE1 for 72 h were compared. Apoptosis was quantified using the Caspase-Glo 3/7 assay, and cell viability was quantified using an MTT assay. Bars represent mean ± SD of triplicate. Statistical significance was determined using Student’s t test. ∗*p* < 0.05 and ∗∗*p* < 0.01.

To identify the genes whose losses induce apoptosis in *KRAS*-mutated NSCLC, we conducted a CRISPR-knockout screen using a crRNA library targeting 746 protein kinase genes. This screen was designed to determine the impact of each kinase on the viability of H23 (*KRAS*-G12C-mutant) cells. We identified 34 genes as potential therapeutic targets because their knockouts reduced cell viability by over 50%. WEE1 knockout was the most effective, significantly reducing cell viability (Figure 1B)*.* Similar results were obtained in H358 (*KRAS*-G12C mutant) cells. Two overlapping genes, WEE1 and Polo-like kinase 1 (PLK1), were among the top 50 genes in both H23 and H358 cells (Figures 1B–1D and S1A).

WEE1 tyrosine kinase is a critical regulator of the G2/M cell cycle checkpoint,^13^^,^^14^^,^^15^ while PLK1 is associated with cell cycle progression via CyclinB1-Cdk1 phosphorylation.^16^ To determine the most appropriate therapeutic target, we evaluated the effects of small interfering RNAs (siRNAs) specific to WEE1 and PLK1 in normal fibroblast cells (IMR-90 and MRC-5). The knockdown of WEE1 did not change the cell viability, while that of PLK1 decreased the viability of both cell lines (Figure S1B). In support of this result, treatment with volasertib, a PLK1 inhibitor, inhibited the growth of both cell lines, whereas adavosertib, a WEE1 inhibitor, had a minimal inhibitory effect (Figure S1C). These results suggest that WEE1 is an optimized therapeutic target with relatively few side effects for *KRAS*-mutated NSCLC.

Given that 30%–40% of patients with *KRAS*-mutated lung cancer have co-existing *TP53* mutations and poor prognoses, we evaluated the effect of WEE1 downregulation in *KRAS*-mutated NSCLC cell lines with or without *TP53* mutations. Importantly, WEE1 knockdown suppressed the growth of *KRAS*-mutated lung cancer cells with *TP53* mutations while having a minimal effect on *TP53* wild-type *KRAS*-mutated NSCLC cell lines (Figures 1E–1G). WEE1 knockdown significantly increased cleaved caspase (c-caspase)-3/7 activity in *KRAS*-mutated NSCLC cell lines with *TP53* mutations (Figure 1H), indicating that apoptosis was induced in these cells. In addition, CRISPR-Cas9 screening showed that WEE1 knockout did not reduce the viability of A549 cells (*TP53* wild type) (Figure S1D). These findings indicate that WEE1 is a potential target for enhancing apoptosis in *KRAS*-mutated NSCLC cell lines with *TP53* mutations.

To understand the relationship between *TP53* mutations and prognosis in patients with *KRAS*-mutated lung cancer, we analyzed clinical data from patients with *KRAS*-mutated lung cancer in The Cancer Genome Atlas using a bioinformatics approach. Among the patients, 115 samples corresponded to the *TP53*-mutant status, and 225 samples contained wild-type *TP53*. Importantly, patients with *KRAS*-mutated lung cancer with wild-type *TP53* had a more optimized prognosis even after 120 months. In contrast, *TP53* mutations were associated with low survival rates of patients with *KRAS*-mutated cancer (Figure S2).

### Drug screening identifies WEE1 inhibitors as potent enhancers of apoptosis

**Figure 2:**
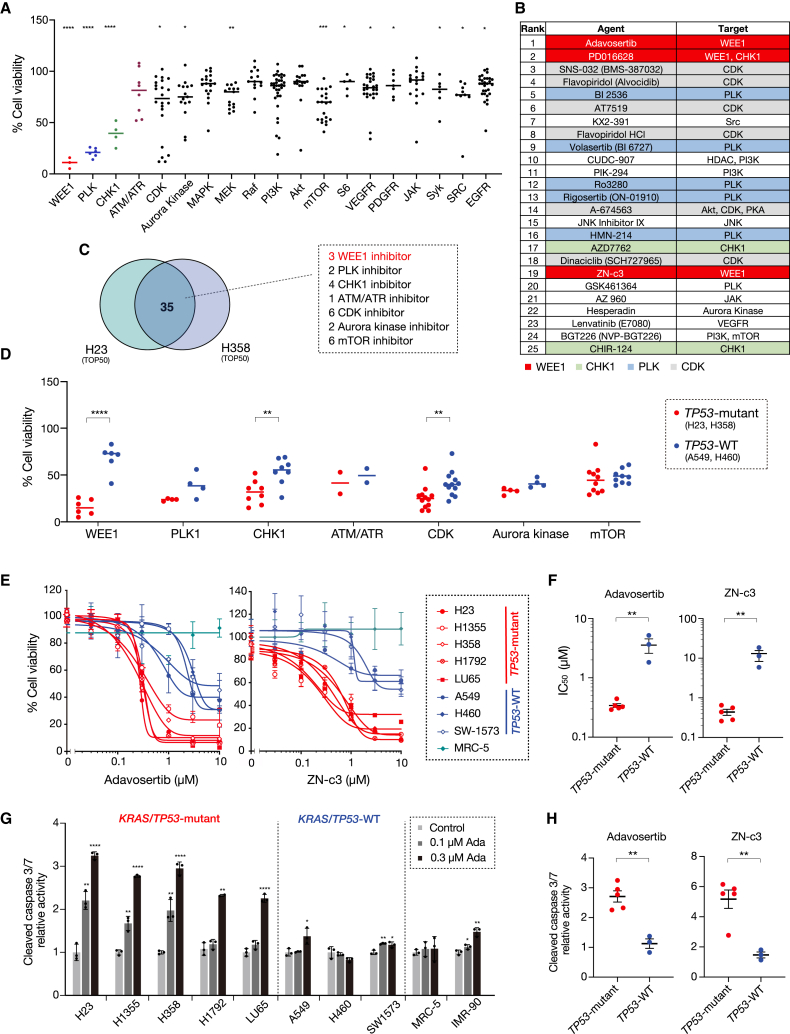
Drug screening identifies WEE1 inhibitors as potent enhancers of apoptosis (A) H23 and H358 cells were treated with each compound (1 μM) in the library. Cell viability was assessed using an MTT assay at 72 h. An overview of the growth inhibition of H23 cells by various pathway inhibitors is provided. Bars represent mean ± SD. Each inhibitor’s efficacy was compared to the control group using Student’s t test. ∗*p* < 0.05, ∗∗*p* < 0.01, ∗∗∗*p* < 0.001, and ∗∗∗∗*p* < 0.0001. (B) The top 25 agents that enhanced the growth inhibition of H23. Red clusters represent WEE1 inhibitors, green clusters CHK1 inhibitors, blue clusters PLK inhibitors, and gray clusters CDK inhibitors. (C) Venn diagram showing the top 50 agents that enhance growth inhibition of H23 and H358 cells. (D) Growth inhibition by various inhibitors was compared between *TP53*-mutant H23 and H358 cells and *TP53*-WT A549 and H460 cells. Bars represent mean ± SD. Statistical significance was determined using Student’s t test. ∗∗*p* < 0.01 and ∗∗∗∗*p* < 0.0001. (E and F) H23, H1355, H358, H1792, LU65, A549, H460, SW1573, and MRC-5 cells were treated with indicated concentrations of adavosertib or ZN-c3 (E). IC_50_ was assessed by an MTT assay at 72 h and compared between the *TP53*-mutant and *TP53*-WT *KRAS*-mutated cell groups, as shown in (F). Bars represent mean ± SD of triplicate. Statistical significance was determined using Student’s t test. ∗∗*p* < 0.01. (G and H) H23, H1355, H358, H1792, LU65, A549, H460, SW1573, MRC-5, and IMR-90 cells were treated with adavosertib at the indicated concentrations for 48 h (G). Apoptosis was quantified using the Caspase-Glo 3/7 assay and compared for *TP53*-mutant and *TP53*-WT KRAS-mutated cell groups, as shown in (H). Bars represent mean ± SD of triplicate. Statistical significance was determined using Student’s t test. ∗*p* < 0.05, ∗∗*p* < 0.01, ∗∗∗*p* < 0.001, and ∗∗∗∗*p* < 0.0001.

To identify drugs that can trigger apoptosis in *KRAS*-mutated NSCLC cells with *TP53* mutations, we performed drug screening in H23 and H358 (*TP53-*mutant) cells using a custom library of kinase inhibitors consisting of 432 compounds. Consistent with the results of our CRISPR-Cas9 screening, WEE1 inhibitors were the most effective targets in the library, inhibiting cell viability by more than 70% in H23 and H358 cells (Figures 2A and S3A). Of the top 50 hit compounds in H23 and H358 cells, 35 overlapping compounds were identified, including 3 WEE1 inhibitors, 2 PLK inhibitors, 4 checkpoint kinase 1 (CHK1) inhibitors, 6 cyclin-dependent kinase (CDK) inhibitors, 2 Aurora kinase inhibitors, and 6 inhibitors of the mammalian target of rapamycin pathway (Figures 2B, 2C, and S3B).

We also conducted drug screening in *TP53* wild-type *KRAS*-mutated NSCLC cell lines (A549 and H460). However, several compounds, including WEE1, CHK1, and CDK inhibitors, did not effectively suppress cell viability compared to *TP53*-mutant cells (Figure 2D). The WEE1 inhibitor adavosertib is currently being evaluated in clinical trials.^17^ While showing promising clinical benefits, it has limited tolerability, probably owing to its poor kinase selectivity. Recently, ZN-c3 (azenosertib), which has higher WEE1 selectivity, was developed by Zentalis Pharmaceuticals,^18^ and a phase 1 dose-escalation trial showed improved safety results compared to those of adavosertib.^19^

To evaluate the efficacy of the WEE1 inhibitors, we measured the half-maximal inhibitory concentration (IC_50_) after 72 h of treatment. Our results showed that each of the WEE1 inhibitors, adavosertib and ZN-c3, inhibited cell viability in a dose-dependent manner in all *KRAS*-mutated NSCLC cell lines with *TP53* mutations tested. The efficacies of adavosertib and ZN-c3 were similar, whereas IC_50_ values were lower in the *TP53*-mutant cell lines than in the *TP53* wild-type cell lines (Figures 2E and 2F). Similar results were obtained in a cell growth assay after another week of treatment (Figure S4A). Furthermore, all *TP53*-mutant cell lines showed remarkable activation of the apoptosis marker c-caspase-3/7, whereas no changes were observed in the *TP53* wild-type cell lines (Figures 2G , 2H, and S4B). Furthermore, these WEE1 inhibitors were also effective in cell lines with other *KRAS* mutation variants, such as H1573 (*KRAS*-G12A, *TP53* mutant) and Calu6 (*KRAS*-Q61K, *TP53* mutant) (Figure S4C). These findings suggest that *TP53*-mutant *KRAS*-mutated NSCLC cells are more sensitive to WEE1 inhibitors than *TP53* wild-type *KRAS*-mutated NSCLC cells.

### *TP53* mutation increases vulnerability to WEE1 inhibition of *KRAS*-mutated NSCLC

**Figure 3:**
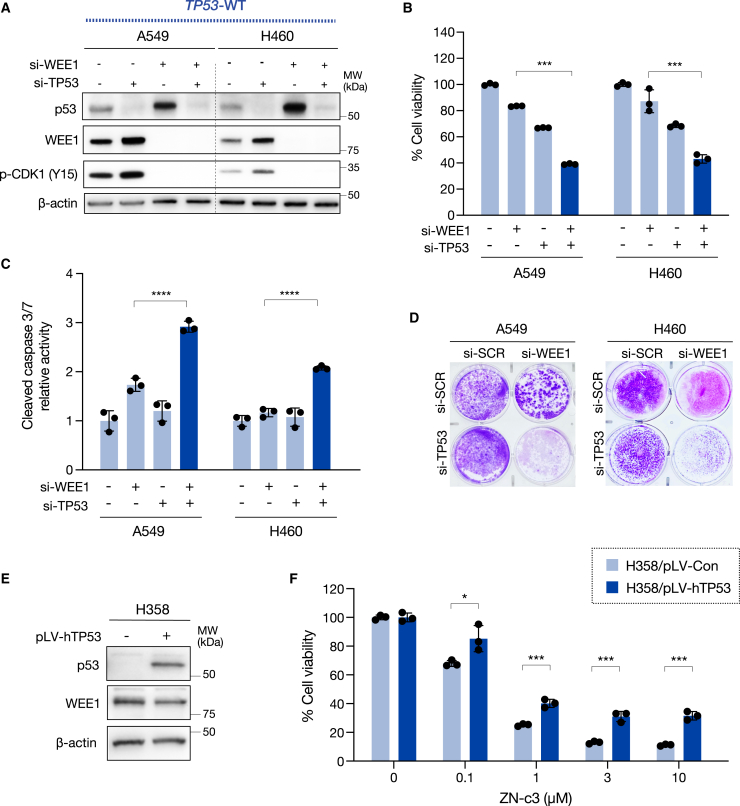
*TP53* mutation increases vulnerability to WEE1 inhibition in *KRAS*-mutated NSCLC (A) A549 and H460 cells were transfected with siRNAs targeting TP53 for 48 h, followed by transfection with siRNAs targeting WEE1 for 72 h. Cell lysates were analyzed by western blotting with the indicated antibodies. (B) The cell viability was assessed using an MTT assay. Bars represent mean ± SD of triplicate. Statistical significance was determined using Student’s t test. ∗∗∗*p* < 0.001. (C) Apoptosis was quantified using the Caspase-Glo 3/7 assay. Bars represent mean ± SD of triplicate. Statistical significance was determined using Student’s t test. ∗∗∗∗*p* < 0.0001. (D) Cell growth was analyzed after 7 days of si-WEE1 treatment using crystal violet staining. (E) H358 cells were transfected with pLV-hTP53, and cell lysates were analyzed with western blotting using the indicated antibodies. (F) H358/pLV-TP53 cells were treated with ZN-c3 at the indicated concentrations for 72 h. Cell viability was assessed using an MTT assay. Bars represent mean ± SD of triplicate. Statistical significance was determined using Student’s t test. ∗*p* < 0.05 and ∗∗∗*p* < 0.001.

To establish a causal relationship between *TP53* and sensitivity to WEE1 inhibition, we depleted *TP53* in *TP53* wild-type *KRAS*-mutated lung cancer cell lines (A549 and H460) using *TP53-*specific siRNA. After 48 h, we further depleted *WEE1* using a WEE1-specific siRNA for an additional 72 h. The knockdown of *WEE1* led to increased p53 levels, suggesting that WEE1 may suppress p53 expression through an unknown mechanism. However, we confirmed that combination treatment with si-WEE1 and si-TP53 successfully suppressed the expression of both genes (Figure 3A). Remarkably, the knockdown of *TP53* and *WEE1* inhibited cell growth by over 50% (Figure 3B) and greatly increased c-caspase-3/7 activity in A549 and H460 cells (Figure 3C). Similar results were obtained in the cell growth assay after an additional week of treatment (Figure 3D). Subsequently, we expressed wild-type p53 using p-LV-hTP53 (pLV[Exp]-EGFP:T2A:Puro-EF1A>hTP53) in H358 cells, which harbor a *TP53* homozygous deletion (Figure 3E). We found that p53 expression in H358 cells significantly attenuated their sensitivity to the WEE1 inhibitor ZN-c3 (Figure 3F). These data suggest that the loss of p53 expression could increase vulnerability to WEE1 inhibition in *KRAS*-mutated NSCLC.

### WEE1 inhibition decreased the expression of CHK2 and Rad51 in *KRAS*-mutant NSCLC harboring *TP53* mutations

**Figure 4:**
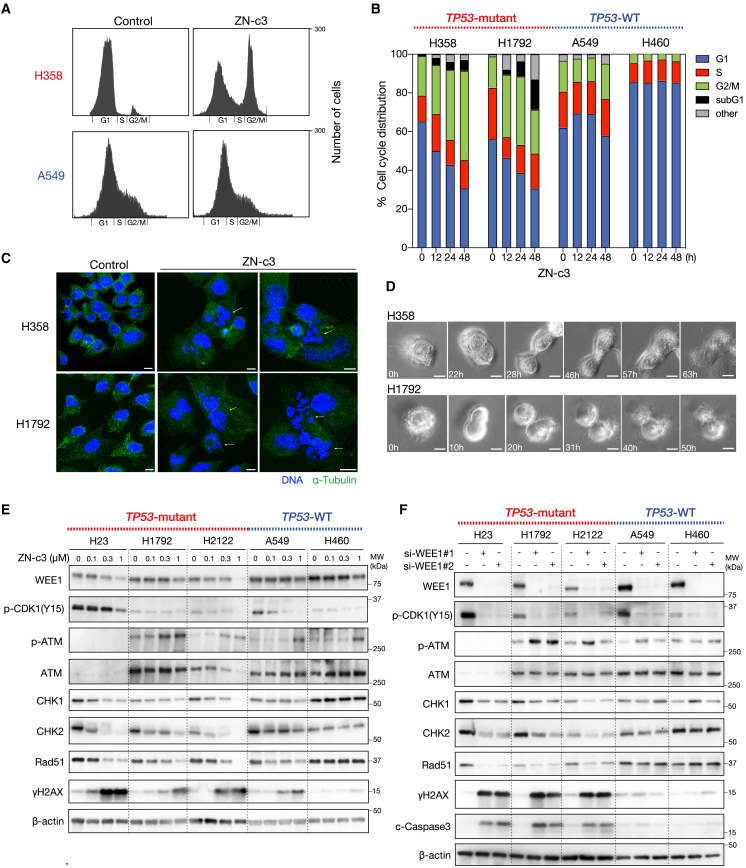
WEE1 inhibition induces mitotic catastrophe in *TP53*-mutant *KRAS*-G12C cells (A and B) H358, H1792, A549, and H460 cells were treated with ZN-c3 (0.5 μM) and subjected to cell cycle analysis using Deep Blue staining at the indicated times. (C) H358 and H1792 cells were treated with 1 μM ZN-c3 for 48 h, subsequently fixed, and stained for α-tubulin (green) via immunofluorescence and for DNA with DAPI (blue). Mitotic catastrophe development was evaluated by analyzing nuclear morphology with a confocal microscope. Representative images of the formation of multinucleated cells are shown (white arrows). Scale bar: 10 μm. (D) Live-cell imaging of H358 and H1792 cells treated with 1 μM ZN-c3 was conducted and continuously monitored through microscopy. Scale bar: 10 μm. (E) H23, H1792, H2122, A549, and H460 cells were treated with ZN-c3 at the indicated concentrations for 48 h. Cell lysates were analyzed using western blotting with the indicated antibodies. (F) H23, H1792, H2122, A549, and H460 cells were transfected with siRNAs targeting WEE1 for 48 h. Cell lysates were analyzed using western blotting with the indicated antibodies.

As WEE1 regulates the G2/M cell cycle checkpoint,^13^ we first performed a cell cycle assay using Deep Red staining and flow cytometry. The results revealed that ZN-c3 induced G2/M cell-cycle arrest in *TP53*-mutant H358 and H1792 cells but not in *TP53* wild-type A549 and H460 cells (Figures 4A and 4B). Then, using confocal microscopy, we observed the formation of multinucleated cells due to incomplete cell division in H358 and H1792 cells treated with ZN-c3 (Figure 4C). In addition, a timelapse of live-cell imaging on H358 and H1792 revealed that the cells were unable to appropriately separate and subsequently either burst or formed multinucleated cells within 72 h (Figure 4D; Videos S1 and S2). These results suggest that WEE1 inhibition induces mitotic catastrophe. To further investigate the molecular mechanism underlying the increased sensitivity of *KRAS*-mutated cells with *TP53* mutations to WEE1 inhibition, we assessed the DDR pathway using western blot analysis. DNA damage sensors ATM and ATR activate downstream effectors CHK1 and CHK2 in response to DNA damage.^20^ These proteins regulate DNA repair through the BRCA1 and Rad51 pathways.^21^^,^^22^ Our analysis revealed increased ATM phosphorylation in H1792 and H2122 cells upon treatment with WEE1 inhibitors. Importantly, the expression of CHK2 decreased remarkably in *TP53*-mutant cells but remained unchanged in *TP53* wild-type cells (Figures 4E and S5). This suggests that WEE1 regulates the expression or stabilization of CHK2. Furthermore, the expression of Rad51 decreased notably, whereas that of CHK1, which is located upstream of Rad51, decreased (Figures 4F and S5). *TP53*-mutant cells also showed robust induction of γ-H2AX, indicating that DNA double-strand break repair was not functional in these cells. Similar results were obtained using two independent siRNA oligos targeting WEE1 (Figure 4D).

Video S1. Live-cell imaging of H1792 cells treated with 1 μM ZN-c3, related to Figure 4D

Video S2. Live-cell imaging of H358 cells treated with 1 μM ZN-c3, related to Figure 4D

These findings suggest that WEE1 plays an important role in the DNA repair system through CHK2 and Rad51 regulation, as well as in the control of the G2/M cell cycle checkpoint in *KRAS*-mutated lung cancer cells with *TP53* mutations.

### WEE1 inhibitors enhance apoptosis in combination with *KRAS*-G12C inhibitor

**Figure 5:**
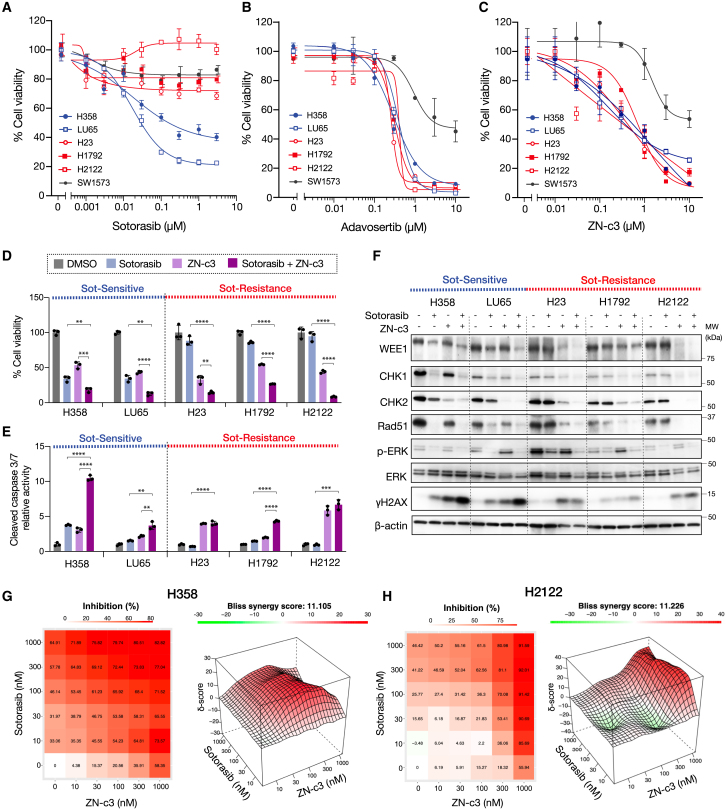
WEE1 inhibitors enhance apoptosis in combination with a *KRAS-*G12C inhibitor (A–C) H358, LU65, H23, H1792, H2122, and SW1573 cells were treated with sotorasib for 72 h at the indicated concentration. The cell viability was assessed using an MTT assay (A). Cells treated with adavosertib or ZN-c3 are shown in (B) and (C). Bars represent mean ± SD of triplicate. (D) H358, LU65, H23, H1792, and H2122 cells were treated with 1 μM sotorasib and/or 1 μM ZN-c3. The cell viability was assessed using an MTT assay at 72 h. Bars represent mean ± SD of triplicate. Statistical significance was determined using Student’s t test. ∗∗*p* < 0.01, ∗∗∗*p* < 0.001, and ∗∗∗∗*p* < 0.0001. (E) Apoptosis was quantified using the Caspase-Glo 3/7 assay at 48 h. Bars represent mean ± SD of triplicate. Statistical significance was determined using Student’s t test. ∗∗*p* < 0.01, ∗∗∗*p* < 0.001, and ∗∗∗∗*p* < 0.0001. (F) Cell lysates were extracted at 48 h and analyzed by western blotting with the indicated antibodies. (G and H) H358 and H2122 cells were treated with ZN-c3 and sotorasib for 72 h at the indicated concentration. Cell viability was assessed using an MTT assay. 2D surface response for cell inhibition and 3D surface Bliss synergy response are shown. Data are presented as mean of triplicates.

Next, we studied the effect of WEE1 inhibition on *KRAS*-G12C-mutated cell lines. Our viability assay showed that LU65 and H358 cells were sensitive to sotorasib and adagrasib (IC_50_ < 1 μM), while H23, H1792, and H2122 showed resistance to sotorasib and adagrasib (IC_50_ > 3 μM) (Figures 5A and S10A). Conversely, each of the WEE1 inhibitors, adavosertib and ZN-c3, reduced cell viability in all cell lines tested (IC_50_: 0.1–1 μM) except for *TP53* wild-type SW1573, indicating that the WEE1 inhibitors may be more effective than sotorasib in *KRAS*-G12C-mutated NSCLC (Figures 5B and 5C).

We evaluated the combined effects of sotorasib and WEE1 inhibitors on cell viability and c-caspase-3/7 activity. Importantly, the dual treatment significantly suppressed cell viability and induced c-caspase-3/7 activity, particularly in sotorasib-sensitive H358 and LU65 cells (Figures 5D, 5E, S6A, and S6B). This effect was confirmed in the treatment of adagrasib (Figures S10B and S10C). Additionally, similar findings were also confirmed using the more specific WEE1 inhibitor Debio0123 (Figures S11A–S11D). In western blot analysis, the DNA-damaged marker γ-H2AX upon treatment with both sotorasib and ZN-c3 (Figure 5F) was increased. In addition, extracellular signal-regulated kinase (ERK) phosphorylation decreased in sotorasib-sensitive H358 and LU65 cells, whereas it slightly decreased in H23 and H1792 cells, suggesting that sotorasib alone was ineffective in these cells (Figures 5F and S6C). Furthermore, we investigated the effects of a combination of sotorasib with the cytotoxic chemotherapy pemetrexed, one of the standard treatments for lung adenocarcinoma, and confirmed that pemetrexed did not enhance the efficacy of sotorasib, unlike the combination of sotorasib and WEE1 inhibitors (Figure S9A).

To further evaluate the drug combination effect, we performed a multidimensional two-drug synergy assay using WEE1 inhibitors combined with sotorasib and assessed the effect using the Bliss independence model. Synergistic effects were considered if the combined effect was more significant than expected for each drug additive (Bliss score > 0). Consequently, the Bliss scores of ZN-c3 plus sotorasib were 11,105 for H358 and 11.226 for H2122 cells (Figures 5G and 5H), indicating a synergistic effect in both sotorasib-sensitive H358 and sotorasib-resistant H2122 cell lines. Similar synergistic effects were observed in treatment combined with adavosertib, as well as in other cell lines treated with a combination of sotorasib and either ZN-c3 or adavosertib (Figures S7A–S7D).

### Dual treatment suppresses DDR via CHK2 inhibition

**Figure 6:**
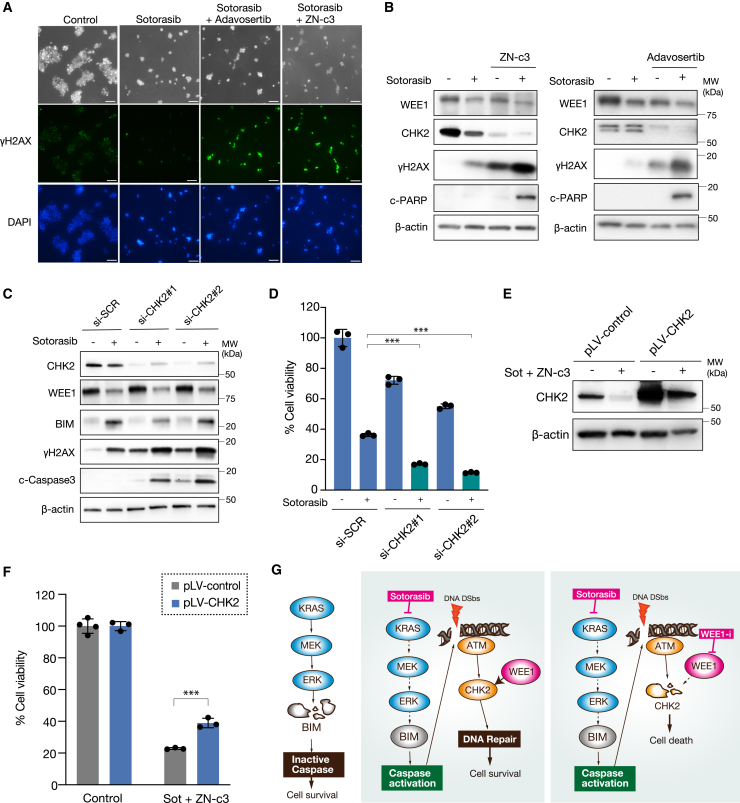
Dual treatment suppresses DNA damage response via CHK2 inhibition (A) Immunofluorescence staining with γH2AX-Alexa 488 and DAPI of H358 cells treated with 1 μM sotorasib, 1 μM sotorasib combined with 1 μM adavosertib, and 1 μM sotorasib combined with 1 μM ZN-c3 for 48 h. Scale bars: 100 μm. (B) H358 cells were treated with 1 μM sotorasib combined with ZN-c3 (1 μM) or adavosertib (1 μM). Cell lysates were extracted at 48 h and analyzed by western blotting with the indicated antibodies. (C) H358 cells were treated with 1 μM sotorasib and transfected with siRNAs targeting CHK2. Cell lysates at 48 h were analyzed by western blotting with the indicated antibodies. (D) Cell viability was assessed using an MTT assay at 72 h. Bars represent mean ± SD of triplicate. Statistical significance was determined using Student’s t test. ∗∗∗*p* < 0.001. (E) H358 cells were transfected with pLV-CHK2, and the cell lysates were analyzed by western blotting with the indicated antibodies. H358/pLV-CHK2 cells were treated with 1 μM sotorasib and 1 μM ZN-c3 for 72 h. (F) Cell viability was assessed using an MTT assay. Bars represent mean ± SD of triplicate. Statistical significance was determined using Student’s t test. ∗∗∗*p* < 0.001. (G) Schematic of the hypothetical roles of WEE1 and CHK2 in *KRAS*-mutated NSCLC cells.

To uncover the underlying mechanisms of the dual treatment, H358 cells were treated with a combination of sotorasib and WEE1 inhibitors, and the level of DNA double-strand breaks was evaluated by analyzing γ-H2AX activity through immunocytochemistry. The results indicated that cells treated with a combination of sotorasib and either adavosertib or ZN-c3 showed strong γ-H2AX activity, while sotorasib treatment alone had no effect (Figure 6A). This was further confirmed by western blotting (Figure 6B). Importantly, WEE1 inhibitors decreased the expression of CHK2, a crucial component in DNA repair (Figure 6B). Therefore, we hypothesized that the reduction in CHK2 expression due to WEE1 inhibition, when combined with sotorasib, leads to apoptosis. The knockdown of CHK2 amplified the induction of γ-H2AX and c-caspase-3 by sotorasib treatment (Figure 6C), and the inhibition of cell growth by sotorasib was significantly enhanced by treatment with si-CHK2 (Figure 6D) The overexpression of CHK2 through the pLV-CHK2 (pLV[Exp]-mCherry:T2A:Hygro-EF1A>hCHEK2) vector partially reversed the effects of the dual treatment on cell viability, thereby confirming the aforementioned result (Figures 6E and 6F). These results suggest that the suppression of CHK2 by WEE1 inhibition exacerbates sotorasib-induced apoptosis in *KRAS*-G12C-mutated cells with *TP53* mutations. The overall findings are summarized in Figure 6G, showing that in the NSCLC case in which *KRAS*-G12C and *TP53* mutations co-exist, sotorasib treatment suppressed ERK, leading to the expression of BIM, which triggered caspase activation through DNA double-strand breaks, whereas WEE1 inhibition reduced the DDR pathway by suppressing CHK2.

### WEE1 inhibition improves the therapeutic efficacy of sotorasib in xenograft models

**Figure 7:**
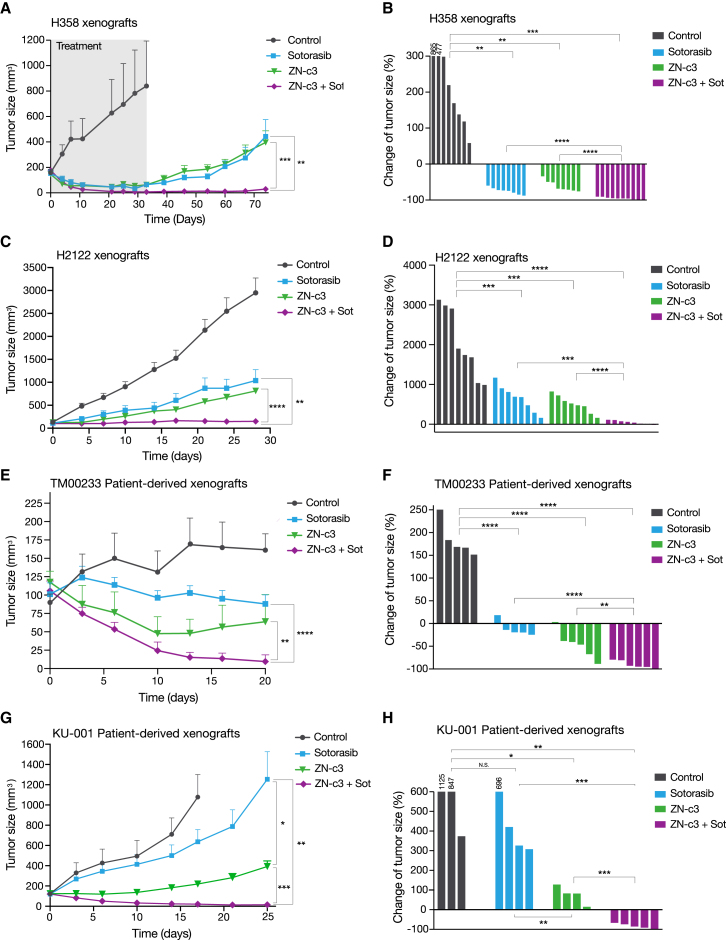
WEE1 inhibition improves the therapeutic efficacy of sotorasib in xenograft models (A) Tumor volumes in mice bearing H358 xenografts treated with vehicle (control: *n* = 8), sotorasib (30 mg/kg: *n* = 8), ZN-c3 (60 mg/kg: *n* = 8), or a combination of ZN-c3 (60 mg/kg) and sotorasib (30 mg/kg) (*n* = 10). (B) Percentage changes in tumor volume after 29 days of treatment in the individual H358 xenografts treated with sotorasib and/or ZN-c3. (C) Tumor volumes in mice bearing H2122 xenografts treated with vehicle (control: *n* = 8), sotorasib (30 mg/kg: *n* = 8), ZN-c3 (60 mg/kg: *n* = 8), or a combination of ZN-c3 (60 mg/kg) and sotorasib (30 mg/kg) (*n* = 10). (D) Percentage changes in tumor volume after 28 days of treatment in the individual H2122 xenografts treated with sotorasib and/or ZN-c3. (E) Tumor volumes in mice bearing TM00233 PDXs treated with vehicle (control: *n* = 5), sotorasib (30 mg/kg: *n* = 5), ZN-c3 (60 mg/kg: *n* = 6), or the combination of ZN-c3 (60 mg/kg) and sotorasib (30 mg/kg) (*n* = 6). (F) Percentage changes in tumor volume after 20 days of treatment in the individual TM00233 xenografts treated with sotorasib and/or ZN-c3. (G) Tumor volumes in KU-001 PDXs treated with vehicle (control: *n* = 3), sotorasib (30 mg/kg: *n* = 4), ZN-c3 (60 mg/kg: *n* = 4), or a combination of ZN-c3 (60 mg/kg) and sotorasib (30 mg/kg) (*n* = 5). (H) Percentage changes in tumor volume after 17 days of treatment with sotorasib and/or ZN-c3. All bars are presented as mean ± SEM of experimental replicates. Significant differences were determined using Student’s t test. ∗*p* < 0.05, ∗∗*p* < 0.01, ∗∗∗*p* < 0.001, and ∗∗∗∗*p* < 0.0001.

We assessed the *in vivo* therapeutic efficacy of the WEE1 inhibitor and the KRAS-G12C inhibitor in mouse xenograft models. Mice with H358 xenografts were treated with sotorasib, ZN-c3, or a combination of ZN-c3 and sotorasib for 32 days (Figure S8A). Notably, both the monotherapy and combination therapy effectively suppressed tumor growth without causing significant toxicity or weight loss (Figures 7A and S8B). Waterfall plot analyses of changes in tumor size showed that tumors treated with the combination regressed by >90% (Figure 7B). We then investigated whether there was any differential tumor regrowth after treatment discontinuation. Notably, the combination therapy prevented tumor regrowth for up to 74 days without treatment, whereas tumors treated with ZN-c3 or sotorasib alone regrew gradually (Figure 7A). In addition, the combination of sotorasib and pemetrexed exhibited weaker efficacy compared to that of the combination of sotorasib and ZN-c3 (Figure S9B), suggesting that targeting of WEE1 is more effective than cytotoxic chemotherapy.

Furthermore, therapeutic efficacy in sotorasib-resistant H2122 cells was evaluated. ZN-c3 monotherapy only mildly suppressed tumor progression compared to the control group (Figure 7C). In contrast to the *in vitro* results, sotorasib monotherapy was as effective as ZN-c3 monotherapy. Importantly, the combination of ZN-c3 and sotorasib consistently suppressed tumor growth and was more effective than the sole administration of either ZN-c3 or sotorasib (Figures 7C and 7D). No significant toxicity or weight loss was observed (Figure S8C).

Next, to reflect a model more similar that of the clinical setting, we evaluated a *TP53*-mutated *KRAS*-G12C patient-derived xenograft (PDX) model obtained from Jaxon (TM00233 patient). We confirmed that sotorasib monotherapy exhibited only mild suppression of tumor progression compared to that of the control group, suggesting an initial resistance to sotorasib. The combined treatment with ZN-c3 nearly completely shrank the tumors (Figures 7E and 7F). Furthermore, we established cell lines and a PDX model (KU-001) from malignant ascites of a patient with *KRAS*-G12C lung cancer at Kanazawa University Hospital. Western blot analysis indicated the absence of p53 expression in the KU-001 cell lines, which is suggestive of a loss of p53 function (Figure S12A). The patient, after being treated with sotorasib, unfortunately progressed to advanced disease and subsequently passed away. Consistent with this clinical observation, treatment of the cell lines with sotorasib showed resistance, whereas treatment with ZN-c3 suppressed cell viability and induced apoptosis. Furthermore, the combination of sotorasib and ZN-c3 was demonstrated to be even more effective (Figures S12B–S12D). Additionally, in the PDX model, sotorasib treatment alone did not suppress the tumor, indicating initial resistance. While ZN-c3 alone had a mild effect, its combination with sotorasib achieved near-complete tumor eradication (Figures 7G and 7H).

Summarily, our findings indicate that the combination of ZN-c3 and sotorasib is a more effective therapeutic strategy for both sotorasib-sensitive and (initially) sotorasib-resistant *KRAS*-G12C-mutated NSCLC with *TP53* mutations.

## Discussion

The development of KRAS-G12C inhibitors such as sotorasib and adagrasib has not led to curative outcomes in most patients with *KRAS*-mutant NSCLC, and those who respond to the treatment often exhibit resistance. Therefore, we employed a dual-screening approach using gene knockouts and drug libraries to identify drugs that could effectively eliminate *KRAS*-mutant NSCLCs. This approach led us to discover a WEE1 inhibitor that strongly induced apoptosis, particularly in *KRAS*-mutant NSCLCs that also harbored *TP53* mutations.

WEE1 plays a crucial role as a protein kinase that regulates the G2/M cell cycle checkpoint, a pivotal point in the cell cycle where DNA damage is assessed before cells enter mitosis.^13^^,^^14^^,^^15^ Because p53 controls the G2/M and G1/S checkpoints, *TP53*-mutant cancers with reduced p53 function may be relatively dependent on the G2/M checkpoint controlled by WEE1.^23^^,^^24^ Therefore, inhibition of WEE1 in *KRAS*-mutated lung cancer cells with *TP53* mutations could lead to mitotic catastrophe, inducing DNA damage and cell death, whereas normal cells with p53 function would be spared. Recent clinical research has demonstrated that adavosertib, a WEE1 inhibitor, improves PFS in patients diagnosed with metastatic cancer with *RAS* and *TP53* mutations.^25^

The effectiveness of WEE1 inhibition in *KRAS-*mutant cancer may be related to replication stress (RS), a type of cellular stress that occurs when the DNA replication machinery encounters obstacles that inhibit DNA replication. This stress can cause the stagnation, collapse, and asymmetry of the replication fork, leading to DNA damage and genomic instability.^26^ RS is a common feature of many types of cancer, including those with *KRAS* mutations.^27^ In contrast, cancer cells adapt to RS by activating the DDR pathways, which promote DNA repair and inhibit apoptosis.^28^ Specifically, *RAS* mutations are frequently associated with the activation of the DDR pathway, as evidenced by elevated DNA damage, activation of DNA damage checkpoints, and cell-cycle arrest.^26^^,^^29^ DDR activation is an intracellular reaction to genotoxic stress that is directly induced by oncogenic *RAS*.^28^ Oncogenic RAS expression leads to an elevation of ATR activity and enhanced dependence on ATR functionality to maintain genomic stability on a per-cell-cycle basis.^30^ Additionally, oncogenic *KRAS* activates CHK1 via the wild-type HRAS and NRAS proteins. Suppression of wild-type H/N-RAS activity can lead to impaired CHK1 function and checkpoint activation, resulting in increased DNA damage.^31^ These findings suggest that *KRAS*-mutant lung cancer cells survive by activating the DDR pathway to evade RS.

WEE1 is not directly involved in DNA damage repair, and its main role is to prevent cells with damaged DNA from entering mitosis and propagating damaged DNA by regulating the cell cycle through the activation of DNA damage checkpoints.^13^^,^^14^ Specifically, when DNA damage occurs, WEE1 is activated by the ATR-CHK1 cascade, which inactivates CDK1 kinase by phosphorylating CDK1 at Tyr15 and inhibiting its entry into mitosis at G2/M.^13^^,^^14^^,^^15^^,^^32^ However, we found that WEE1 inhibition markedly reduced the expression of CHK2 and Rad51, both of which are involved in DNA damage repair (Figures 4E and 4F). Phosphorylation of the C-terminal domain of BRCA2 by CHK1 or CHK2 plays a critical role in the binding of Rad51 to BRCA2 and the subsequent recruitment of Rad51 to sites of DNA damage.^21^ A recent study showed that *KRAS*-mutant cells rely more on the DNA damage repair protein Rad51 than wild-type *KRAS* cells. Depletion of Rad51 in *KRAS-*mutant cells increased the occurrence of DNA double-strand breaks.^33^ These findings suggest that WEE1 is located upstream of the DDR pathway and may play a more direct role in DNA damage repair through CHK2 and Rad51, as well as in cell cycle regulation in *KRAS*-mutant NSCLC cells (Figure S13). WEE1 may be a master regulator of the entire DDR pathway; therefore, WEE1 inhibition has a potent inhibitory effect on *KRAS-*mutant NSCLC cells. Consistent with this concept, our CRISPR-knockout and drug screening studies have shown that targeting WEE1 leads to the highest growth inhibition compared to other DDR factors, including ATM, ATR, CHK1, and CHK2 (Figures 1B and 2A). This study demonstrates the distinct role of WEE1 in the DDR pathway. Further studies are needed to fully elucidate the mechanism by which WEE1 regulates CHK2 and Rad51.

*In vivo* monotherapy with a WEE1 inhibitor resulted in growth inhibition comparable to that of sotorasib in *KRAS*-mutant lung cancer with *TP53* mutations (Figures 7A and 7C). In PDX models, sotorasib alone showed minimum efficacy, while the WEE1 inhibitor resulted in higher growth inhibition compared to that resulting from sotorasib treatment (Figures 7E and 7G). Importantly, we showed that the combination treatment with ZN-c3 and sotorasib resulted in marked tumor regression in three xenograft models, including two PDX models (Figures 7B, 7F, and 7H). While a previous study also showed the effectiveness of the WEE1 inhibitor adavosertib as a monotherapy in *KRAS*-mutant cancer cell lines with *TP53* mutations,^34^ our research extends this understanding by demonstrating enhanced therapeutic benefits when combining KRAS inhibitors with WEE1 inhibitors in both sotorasib-sensitive and -resistant *KRAS-*G12C with *TP53* mutations. The mechanism by which the dual treatment induces apoptosis has yet to be determined. Recent studies have shown that targeted oncogene therapy, which includes inhibitors of EGFR, ALK, KRAS, and BRAF, induces DNA double-strand breaks and activates the DDR pathway in residual tumor cells.^35^ This suggests that combining molecularly targeted drugs with DDR pathway inhibitors may be an effective treatment strategy for driver-mutant lung cancers. In *EGFR-*mutated NSCLC models, tumor cells that survive treatment with an EGFR inhibitor are synthetically dependent on ATM in the DDR pathway, and combined treatment with an ATM kinase inhibitor eradicates these cells *in vivo.*^35^ In the present study, our analysis suggests that sotorasib-induced apoptosis is protected by the activation of the WEE1-CHK2 axis in *KRAS*-mutated cancer cells (Figure 6G). This allowed the cells to survive and may have contributed to early tolerance to sotorasib. Further studies are required to elucidate the detailed mechanisms.

It is well known that *KRAS* mutations often co-exist with other mutations, including those in *TP53*, *KEAP1*, and *STK11*. Recent studies have shown that the *KEAP1* mutation is associated with reduced responsiveness to KRAS-G12C inhibitors, while the *STK11* mutation does not significantly affect the response. Additionally, alterations in DDR pathways, including CHK2, have been suggested to enhance the efficacy of KRAS-G12C inhibitors.^36^ Our study investigated the efficacy of the combination of a KRAS-G12C inhibitor with a WEE1 inhibitor. We found that this combination was effective in cell lines with *LKB1*, *KEAP1*, and *TP53* mutations (H2122 and H23) and *LKB1*- and *TP53*-mutated LU65 cells, as well as the H2122 xenograft model, indicating the potential effectiveness of this treatment strategy regardless of the presence of these mutations. These findings suggest that the combination therapy of KRAS-G12C and WEE1 inhibitors would be effective in patients with *KRAS*-G12C-mutated tumors, including those with concurrent *KEAP1* or *LKB1* mutations.

WEE1 inhibitors have been developed to target various types of cancer, including advanced solid tumors, such as ovarian, endometrial, mesothelioma, breast, colon, pancreatic, and NSCLC.^17^^,^^37^ Historically, adavosertib was evaluated in both preclinical and clinical studies, including its combination with chemotherapy, such as a phase 2 study of adavosertib combined with carboplatin for the treatment of ovarian cancer with *TP53* mutations^17^^,^^38^ and a phase 1 study combined with docetaxel and cisplatin for the treatment of pancreatic cancer.^39^ In PDX models of pancreatic cancer, adavosertib combined with irinotecan or capecitabine significantly inhibited tumor growth, especially in cases with *TP53*-mutant status.^40^ These findings suggest that WEE1 inhibition, particularly in combination with chemotherapy, might be more effective than monotherapy and that *TP53* mutation status could be a predictive biomarker for identifying treatment strategies involving WEE1 inhibitors. There is also ongoing research into adavosertib combined with immunotherapy, such as durvalumab treatment, for advanced solid tumors.^41^ However, a higher incidence of grade ≥3 adverse events, particularly hematological toxicities, has been observed when used in combination with standard treatments.^17^ Consequently, the development of adavosertib for certain conditions, such as ovarian cancer, solid tumors, and uterine serous cancer, including for treatment in combination with durvalumab, was discontinued.^42^ Zentalis Pharmaceuticals developed a next-generation WEE1 inhibitor called ZN-c3,^18^ which showed markedly lower hematological toxicities compared to those observed with adavosertib owing to its higher selectivity for WEE1 inhibition.^19^ In our *in vivo* studies, both ZN-c3 alone and in combination with sotorasib demonstrated high efficacy without notable side effects. Phase 1 clinical trials of ZN-c3 are currently ongoing, targeting patients with solid tumors, ovarian cancer, peritoneal cancer, and breast cancer.

In conclusion, our study suggests that the combination of sotorasib and the next-generation WEE1 inhibitor ZN-c3 is a promising treatment for *KRAS-*G12C-mutated lung cancer with *TP53* mutations. This treatment regimen significantly suppressed tumor regrowth and promoted remission in four xenograft studies involving PDX models. However, the safety and efficacy of this combination therapy should be evaluated and validated in clinical trials.

### Limitations of the study

Our study highlights the promise of combination therapy with WEE1 and KRAS G12C inhibitors in *KRAS*-G12C-mutant NSCLC with *TP53* mutations. However, its efficacy against the acquired resistance of KRAS-G12C inhibitor monotherapy remains to be determined, suggesting that initial concurrent use may be beneficial to anticipate and prevent resistance development. The interaction of this combination with different NSCLC genetic backgrounds and co-mutations requires further elucidation. Furthermore, clinical trials are essential to validate the safety and efficacy of this therapy in a clinical setting, especially considering the historical challenges associated with WEE1 inhibitors. Additionally, its potential effectiveness in other types of *KRAS*-G12C-positive solid tumors with *TP53* mutations, such as colorectal, pancreatic, endometrial, ovarian, and appendiceal cancers, has not been validated. Future studies are needed to explore the applicability and benefits of this treatment approach across a broader spectrum of *KRAS-*G12C-mutant cancers.

## STAR★Methods

### Key resources table

**Table:** 

REAGENT or RESOURCE	SOURCE	IDENTIFIER
**Antibodies**		

WEE1, Rabbit monoclonal	Cell Signaling Technology	Cat#13084; RRID: AB_2713924
p53, Rabbit monoclonal	Cell Signaling Technology	Cat#2527; RRID: AB_10695803
Phospho-cdc2 (Tyr15), Rabbit monoclonal	Cell Signaling Technology	Cat#4539; RRID: AB_560953
phospho-MAPK (Erk1/2) (Thr202/Tyr204), Rabbit monoclonal	Cell Signaling Technology	Cat#4370; RRID: AB_2315112
P44/42 (Erk1/2), Rabbit monoclonal	Cell Signaling Technology	Cat#4695; RRID: AB_390779
Chk1, Mouse monoclonal	Cell Signaling Technology	Cat#2360; RRID: AB_2080320
Chk2, Rabbit monoclonal	Cell Signaling Technology	Cat#6334; RRID: AB_1178526
ATM, Rabbit monoclonal	Cell Signaling Technology	Cat#2873; RRID: AB_2052569
phosopho-ATM, Rabbit monoclonal	Abcam	Cat#ab81292; RRID:AB_1640207
Rad51, Rabbit monoclonal	Cell Signaling Technology	Cat#8875; RRID: AB_2721109
Cleaved Caspase-3, Rabbit monoclonal	Cell Signaling Technology	Cat#9664; RRID: AB_2070042
Cleaved PARP, Rabbit monoclonal	Cell Signaling Technology	Cat#5625; RRID: AB_10699459
Bim, Rabbit monoclonal	Cell Signaling Technology	Cat#2993; RRID: AB_490935
Phosopho-Histone H2A.X, Rabbit polyclonal	Cell Signaling Technology	Cat#2577; RRID: AB_2118010
β-Actin, Rabbit monoclonal	Cell Signaling Technology	Cat#4970; RRID: AB_2223172
α-Tubulin, Rabbit monoclonal	Cell Signaling Technology	Cat#2125; RRID: AB_2619646
Anti-rabbit IgG, HRP-linked antibody	Cell Signaling Technology	Cat#7074; RRID: AB_2099233
Anti-mouse IgG, HRP-linked antibody	Cell Signaling Technology	Cat#7076; RRID: AB_330924

**Biological samples**		

Patient-derived xenograft (PDX) TM00233	The Jackson Laboratory-USA	Cat#TM00233
Patient-derived xenograft (PDX) KU-001	This paper	N/A

**Chemicals, peptides, and recombinant proteins**		

Sotorasib	Selleck Chemicals	Cat#S8830
Adavosertib	MedChemExpress	Cat#HY-10993
ZN-c3 (Azenosertib)	Selleck Chemicals	Cat#E1000
Debio0123	Selleck Chemicals	Cat#S9778
Volasertib	Selleck Chemicals	Cat#S2235
Pemetrexed	Selleck Chemicals	Cat#S5917
Adagrasib	Selleck Chemicals	Cat#S8884
Lipofectamine™ RNAiMAX Transfection Reagent	Invitrogen	Cat#13778150
Lipofectamine™ LTX Reagent with PLUS™ Reagent	Invitrogen	Cat#15338100
Antifade Mounting Medium with DAPI	VECTASHIELD	Cat#H-1500

**Critical commercial assays**		

Cell Cycle Assay Solution Deep Blue	Dojinbo	Cat#341-09601
Caspase-Glo® 3/7 Assay	Promega	Cat#G8091

**Experimental models: Cell lines**		

NCI-H358 (KRAS-G12C, TP53-homo deletion)	ATCC	Cat#CRL-5807; RRID: CVCL_1559
NCI-H23 (KRAS-G12C, TP53-homo M246I)	ATCC	Cat#CRL-5800; RRID: CVCL_1547
NCI-H1355 (KRAS-G13C, TP53-E285K)	ATCC	Cat#CRL-5865; RRID: CVCL_1464
NCI-H1792 (KRAS-G12C, TP53-Splice doner site)	ATCC	Cat#CRL-5895; RRID: CVCL_1495
A549 (KRAS-G12S, TP53-WT)	ATCC	Cat#CRM-CCL-185; RRID: CVCL_0023
NCI-H460 (KRAS-Q61H, TP53-WT)	ATCC	Cat#HTB-177; RRID: CVCL_0459
SW1573 (KRAS-G12C, TP53-WT)	ATCC	Cat#CRL-2170; RRID: CVCL_1720
LU65 (KRAS-G12C, TP53-E11Q)	Japanese Cell Research Bank	Cat#JCRB0079; RRID: CVCL_1392
NCI-H2122 (KRAS-G12C, TP53-Q16L, C176F)	ATCC	Cat#CRL-5985; RRID: CVCL_1531
NCI-H1573 (KRAS-G12A, TP53-R248L)	ATCC	Cat#CRL-5877; RRID: CVCL_1478
Calu6 (KRAS-Q61K, TP53-R196Ter)	ATCC	Cat#HTB-56; RRID: CVCL_0236
MRC-5	ATCC	Cat#CCL-171; RRID: CVCL_0440
IMR-90	ATCC	Cat#CCL-186; RRID: CVCL_0347

**Experimental models: Organisms/strains**		

SHO (Crlj: SHO-Prkdc^scid^Hr^hr^) mice, females, 6weeks old	The Jackson Laboratory, Japan	N/A

**Oligonucleotides**		

*Silencer*®Select siRNA: WEE1	Invitrogen	Cat#S21
*Silencer*®Select siRNA: WEE1	Invitrogen	Cat#S22
*Silencer*®Select siRNA: TP53	Invitrogen	Cat#S605
*Silencer*®Select siRNA: TP53	Invitrogen	Cat#S606
*Silencer*®Select siRNA: CHK2	Invitrogen	CatS22119
*Silencer*®Select siRNA: CHK2	Invitrogen	Cat#S22120
*Silencer*®Select siRNA: PLK1	Invitrogen	Cat#S448
*Silencer*®Select siRNA: PLK1	Invitrogen	Cat#S449
*Silencer*®Select Negative Control siRNA#1	Invitrogen	Cat#4390843
Dharmacon Edit-R™ synthetic sgRNA libraries	Horizon Discovery	Cat#GA-005100-01

**Recombinant DNA**		

pLV[Exp]-EGFP:Puro-EF1A > ORF_stuffer	VectorBuilder	Cat#VB010000-9389rbj
pLV[Exp]-mCherry/Hygro-EF1A > ORF_stuffer	VectorBuilder	Cat#VB010000-9293ufr
pLV[Exp]-EGFP:T2A:Puro-EF1A > hTP53	VectorBuilder	Cat#VB900006-7720qtw
pLV[Exp]-mCherry:T2A:Hygro-EF1A > hCHEK2	VectorBuilder	Cat#VB900137-9902ptw
LentiV_Cas9_puro	Addgene	Cat#108100

**Software and algorithms**		

GraphPad Prism Ver. 8.0	GraphPad Software	https://www.graphpad.com/features
TCGA (The Cancer Genome Atlas)	National Cancer Institute	https://www.cancer.gov/ccg/research/genome-sequencing/tcga
SynergyFinder	SynergyFinder open sourse	https://synergyfinder.fimm.fi/

### Resource availability

#### Lead contact

Further information and requests for resources and reagents should be directed to and will be fulfilled by the lead contact, Koji Fukuda (kfukuda@staff.kanazawa-u.ac.jp).

#### Materials availability

This study did not generate new unique reagents.

#### Data and code availability

This paper does not contain any original code. The datasets generated and/or analyzed during this study are available from the Lead Contact for the purposes of reanalyzing the data reported in this paper. Any additional information required to reanalyze the data reported in this paper is available from the Lead Contact upon request.

### Experimental model and study participant details

#### Cell lines and cell cultures

NSCLC human-derived cell lines were obtained from the American Type Culture Collection (ATCC) and Japanese Cell Research Bank (Key resources table). The summary of *KRAS* and *TP53* mutations in the cell lines is shown in Table S1. All cell lines were maintained in Roswell Park Memorial Institute (RPMI) media supplemented with 10% fetal bovine serum, penicillin (100 U/mL), and streptomycin (50 μg/mL) and incubated at 37C with 5% CO2. All cell lines were tested and authenticated by short tandem repeat profiling (DNA fingerprinting) and routinely tested for mycoplasma species before any experiments were performed.

#### Mouse models

This research was carried out in strict accordance with the recommendations of the Guide for the Care and Use of Laboratory Animals of the Ministry of Education, Culture, Science, and Technology in Japan. The protocol was approved by the Committee on the Ethics of Experimental Animals and the Advanced Science Research Center, Kanazawa University, Kanazawa, Japan (approval number AP-153499). Female SHO mice (6 weeks old) were obtained from The Jackson Laboratory, Japan, Patient-derived xenograft (PDX) TM00233 female NGS mice (6 weeks old) were obtained from, female B6 FVBF1/J (6 weeks) and female NSG mice (6 weeks) were obtained from The Jackson Laboratory-USA JACKSON LABORATORY, and housed in accredited facilities under pathogen-free conditions. Additional information on experimental methods in next section.

### Methods details

#### Cell-viability assay

Cells were seeded at 4,000 cells/well in 96-well plates and incubated overnight. The cells were treated with the indicated compounds for 72 h. Cell viability was determined using the MTT assay (Sigma-Aldrich), and the absorbance was measured using an iMark Microplate Absorbance Reader (Bio-Rad). The percentage of cell viability was calculated relative to that of the untreated control or baseline cells. The IC_50_ values were calculated using a nonlinear regression model with a sigmoidal dose-response curve using GraphPad Prism 8 (GraphPad Software, La Jolla, CA, USA). Combined effects were analyzed using SynergyFinder (https://synergyfinder.org/).

#### Western blotting

The cells were washed with PBS (Gibco) and lysed on ice using RIPA buffer (Thermo Fisher Scientific) supplemented with a protease and phosphatase inhibitor cocktail (P8340 and P0044; Sigma-Aldrich Corporation, St. Louis, MO, USA), and the cell extracts were collected. Equal amounts of proteins (20 μg) were electrophoresed on polyacrylamide gels (Mini-PROTEAN TGX Precast Gels: Bio-Rad, Hercules, CA, USA) and transferred to polyvinylidene difluoride membranes (Immun-Blot PVDF Membrane; Bio-Rad). The membranes were then incubated with StartingBlock T20 (TBS) Blocking Buffer (Thermo Fisher Scientific) for 1 h at room temperature and incubated in primary antibodies (1:1000) overnight at 4°C, and horseradish peroxidase-conjugated secondary antibody (#7074) (1:2000 dilution; Cell Signaling Technology) for 1 h at room temperature. All antibodies were diluted with 5% (w/v) BSA (Sigma-Aldrich)/Tris-buffered saline (TBS) with 0.1% (v/v) TWEEN 20 (TBS-T; Sigma-Aldrich), and the membranes were washed with TBS-T between each step three times for 10 min each. Immunoreactive bands were visualized using SuperSignal West Dura Extended Duration Substrate (Thermo Fisher Scientific). Chemiluminescent signals were measured using a FUSION-SOLO Chemiluminescence Imaging System (Vilber Lourmat, Marne-la-Vallée, France). All the blots were obtained from the same experiment and processed in parallel. The data for the full membrane are shown in Figure S14.

#### CRISPR-Cas9 gene editing

Cas9-expressing cells were generated using Cas9 nuclease-expressing lentiviral particles (LentiV_Cas9_puro; #108100; Addgene). Dharmacon Edit-R synthetic sg RNA libraries (Horizon Discovery, Waterbeach, UK) were used for CRISPR-KO screening, and the procedure was performed according to the manufacturer’s instructions.

#### Apoptosis assay

Cells were seeded at 4,000 cells/well in 96-well plates and incubated overnight. The cells were treated with the indicated compounds for 72 h. Apoptosis was quantified using the Caspase-Glo 3/7 Assay (Promega, Madison, WI, USA) according to the manufacturer’s instructions. Luminescence was measured using a Fluoroskan Ascent FL Microplate Fluorometer and Luminometer (Thermo Fisher Scientific). Cell viability was quantified simultaneously using the CellTiter-Glo 2.0 Cell Viability Assay (Promega). Caspase 3/7 levels were normalized to the cell viability.

#### Cell cycle assay

Cells were seeded in 6-well plates at 30–50% confluence and treated with the indicated agents the following day. After the indicated time points, the cells were harvested and stained with Cell Cycle Assay Solution Deep Blue (Dojinbo) for 15 min at room temperature. The stained cells were analyzed by flow cytometry using an SH800 cell sorter (Sony).

#### Immunofluorescence staining

The cells cultured in chamber slides were fixed with ice-cold 100% methanol (FUJIFILM Wako) for 10 min at −20°C. The cells were then permeabilized with 0.25% (v/v) Triton X-100 (Sigma-Aldrich) diluted in PBS for 10  min, followed by blocking with 5% (w/v) BSA/PBS for 30 min at room temperature. Subsequently, the cells were incubated overnight at 4°C with primary antibodies (1:100), followed by incubation with Alexa Fluor 488-conjugated secondary antibody (#4412, 1:1000; Cell Signaling Technology) for 1 h at room temperature. All antibodies were diluted in 5% (w/v) BSA/PBS. Finally, the nuclei were counterstained with DAPI (4′,6-diamidino-2-phenylindole) using VECTASHIELD Antifade Mounting Medium with DAPI (Vector Laboratories, Burlingame, CA, USA). Mitotic catastrophe development was observed using Leica TCS SP8 MP (Leica). γ-H2AX activity was evaluated using ECLIPSE Ti2 (Nikon).

#### Live cell imaging

Cells were seeded at 3,000 cells/well in 96-well plates and incubated overnight. The cells were treated with the indicated compounds for 72 h. Timelapse of live cell imaging was monitored using ECLIPSE Ti2 (Nikon).

#### siRNA transfections

*Silencer*Select siRNAs for WEE1(s21, s22), TP53 (s605, s606), CHK2 (s22119, s22120), and *Silencer* Select Negative Control siRNA #1 (#4390843) were purchased from Thermo Fisher Scientific. Smart-pool Human WEE1 siRNAs were purchased from Dharmacon. Cells were transfected with siRNAs by reverse transfection using Lipofectamine RNAiMAX Transfection Reagent (Invitrogen, Waltham, MA, USA) according to the manufacturer’s instructions. Gene knockdown was confirmed by western blotting.

#### Generation of cDNA-expressing cell lines

The following vectors were purchased from VectorBuilder: pLV[Exp]-EGFP:T2A:Puro-EF1A > hTP53[NM_000546.5], the control vector pLV[Exp]-EGFP:Puro-EF1A > ORF_stuffer, pLV[Exp]-mCherry:T2A:Hygro-EF1A > hCHEK2[NM_001005735.2], and the control vector pLV[Exp]-mCherry/Hygro-EF1A > ORF_stuffer. Cells were transfected using Lipofectamine LTX Reagent with PLUS Reagent according to the manufacturer’s instructions. Gene expression was confirmed via western blotting.

#### Xenograft mouse studies

To establish tumors, H358 and H2122 cells (5.0 × 10^6^ cells) suspended in 50% (v/v) Matrigel (Corning, New York, NY, USA)/Hanks’ balanced salt solution (Gibco) were subcutaneously injected into both flanks of 6-week-old female SHO mice (Crlj: SHO-Prkdc scidHr h). Once the average tumor volume reached approximately 100 mm^3^, the mice were randomly divided into four groups and treated with sotorasib (30 mg/kg/day), ZN-c3 (60 mg/kg/day), a combination of sotorasib and ZN-c3, or control by oral gavage for 5 days per week for 3–5 weeks. Sotorasib was dissolved in 0.5% methylcellulose and 1% Tween 80, whereas ZN-c3 was dissolved in 5% DMSO, 40% PEG 400, and 5% Tween 80. Tumor size and body weight were measured twice per week, and tumor volumes (mm^3^) were calculated using the following formula: [1/2 × length (mm) × width (mm)].^2^ All animal experiments were performed in accordance with the Guide for the Care and Use of Laboratory Animals of the Ministry of Education, Culture, Sports, Science, and Technology, Japan. The study protocol was approved by the Ethics Committee on the Use of Laboratory Animals and Advanced Science Research Center, Kanazawa University, Kanazawa, Japan (approval no. AP-173867).

#### Establishment of patient-derived tumor xenografts

The *KRAS*-G12C patient-derived xenograft (PDX) model was obtained from Jaxon (TM00233 patient). After the tumor reached 1.5 cm in diameter, the mice were euthanized. The tumor was dissected into small specimens (3 mm × 3 mm × 3 mm) and reimplanted in 6-week-old female SHO mice (Crlj: SHO-Prkdc scidHrh). Malignant ascites were collected from a patient with *KRAS-*G12C mutant NSCLC at Kanazawa University Hospital. Tumor fragments obtained from this specimen were used for establishing the PDX models by subcutaneously implanting them into 6-week-old female SHO mice (Crlj: SHO-Prkdc scidHr h). Once the tumor reached 1.5 cm in diameter, the mice were euthanized. The tumor was dissected into small specimens (3 mm × 3 mm × 3 mm) and reimplanted into additional SHO mice for further study. All methods adhered to the guidelines of our institutional Animal Research Committee (Kanazawa University).

### Quantification and statical analysis

Data from cell viability assay, apoptosis assay, micronuclei assay were expressed as means ± standard deviation (SD) and tumor progression in animal studies as means ± standard error (SE), respectively. The statistical significance of differences was analyzed using with GraphPad Prism Ver. 8.0 with *p* value less than 0.05 considered statistically significant (ns > 0.05, ∗*p* < 0.05, ∗∗*p* < 0.01, ∗∗∗*p* < 0.001, ∗∗∗∗*p* < 0.0001).

## Supplemental information

Document S1. Figures S1–S14 and Table S1

Data S1. Comprehensive list of CRISPR-knockout crRNA, related to Figure 1

Data S2. Comprehensive list of drug targets, related to Figure 2

Document S2. Article plus supplemental information
