# Supplementary material for: Targeting WEE1 enhances the antitumor effect of KRAS-mutated non-small cell lung cancer harboring TP53 mutations
Source: Cell Rep Med. 2024 May 21;5(6):101578. doi: 10.1016/j.xcrm.2024.101578 (PMC11228449; doi:10.1016/j.xcrm.2024.101578)
Supplement: Document S1. Figures S1–S14 and Table S1 [file mmc1.pdf]

Cell Reports Medicine, Volume *5*

Supplemental information

Targeting WEE1 enhances the antitumor effect of *KRAS*-mutated non-small cell lung cancer harboring *TP53* mutations

Koji Fukuda, Shinji Takeuchi, Sachiko Arai, Shigeki Nanjo, Shigeki Sato, Hiroshi Kotani, Kenji Kita, Akihiro Nishiyama, Hiroyuki Sakaguchi, Koshiro Ohtsubo, and Seiji Yano

Figure S1

A

	H23		H358	
Rank	Gene	Viablity (%)	Gene	Viablity (%)
1	WEE1	18.2	WEE1	46.8
2	PLK1	22.3	PLK1	52.7
3	PRKAR1B	29.6	NME4	61.6
4	EFNA1	38.4	HSPB8	68.7
5	PIP5K1A	40.5	RPS6KB2	68.8
6	CHKA	40.9	NME2	68.9
7	CSNK1D	41.0	RPS6KC1	69.1
8	XYLB	42.0	MPP5	69.6
9	NME1	42.7	GUCY2F	70.9
10	PKDCC	44.3	GK5	71.4
11	CALM2	44.5	TAB3	72.3
12	NEK10	45.0	EPHB3	72.4
13	ACVR2A	45.0	MARK2	72.6
14	SGK494	45.0	ZAK	73.1
15	NMRK2	45.2	TTK	73.1
16	MST1R	45.5	FASTKD2	73.2
17	PRKAR1A	46.2	MAP3K14	73.3
18	EFNA5	46.5	RPS6KA6	73.5
19	COASY	46.8	PFKFB1	73.5
20	ERN2	47.2	FER	73.8

B

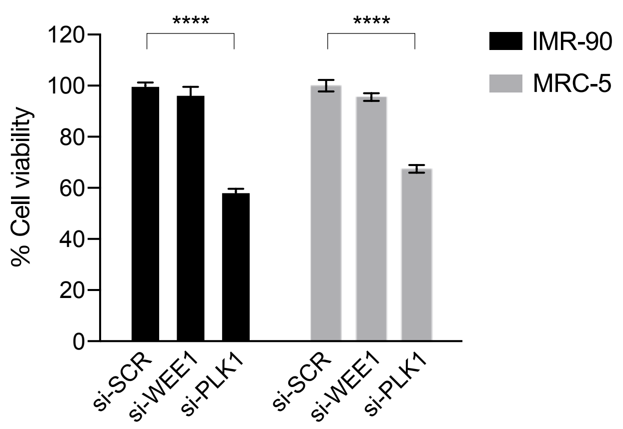

C

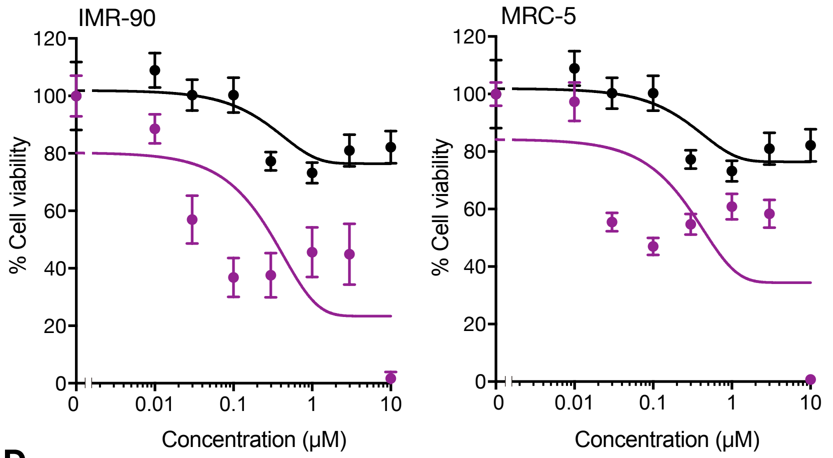

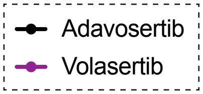

IC_{50} (μM)	MRC-5	IMR-90
Adavosertib	> 10	> 10
Volasertib	0.502	0.33

D

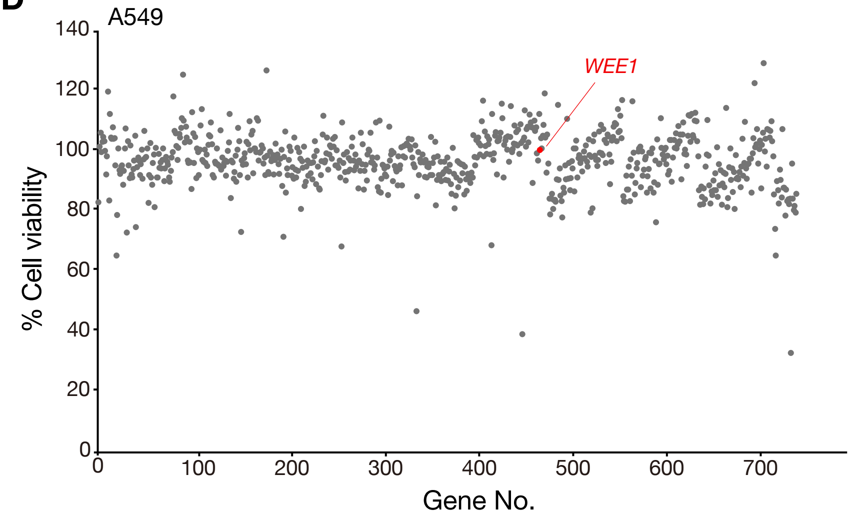

Figure S1. Effect of WEE1 inhibition, related to Figure 1
(A) The top 20 genes that suppress the growth inhibition of H23 and H358 are shown. (B) Cell viability of IMR-90 and MRC-5 cells transfected with siRNAs targeting WEE1 or PLK1 for 72 hours. Cell viability was quantified by MTT assay. Bars represent mean ± SD of triplicate. Statistical significance was determined using Students t test. ****p < 0.0001. (C) IMR-90 and MRC-5 cells were treated with adavosertib or volasertib at the indicated concentration. IC was assessed by MTT assay at 72 hours. Bars represent mean ± SD of triplicate.(D) A549 cells were expressed with CAS9 and treated with a crRNA library for seven days. Cell viability was assessed by MTT assay.

Figure S2

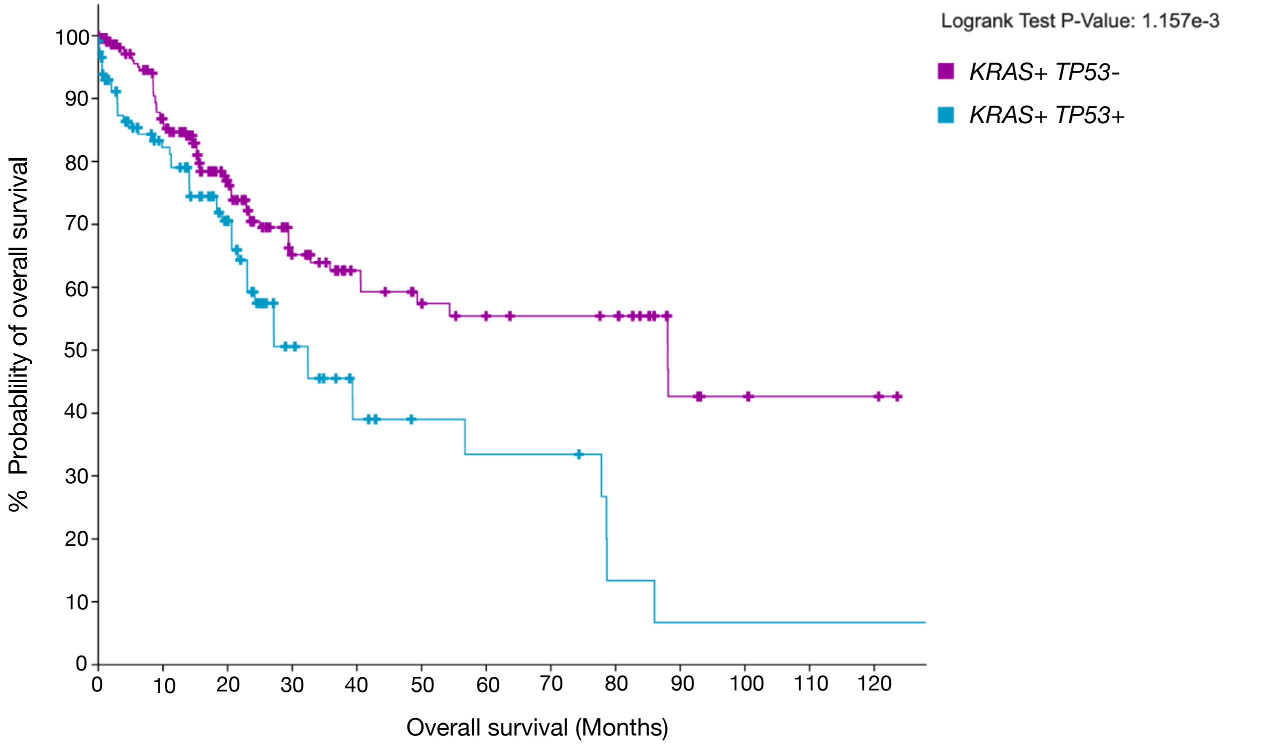

	Number of Cases, Total	Number of Events	Median Months Overall (95% CI)
KRAS+ TP53-	**225**	**64**	**88.07 (49.35 - NA)**
KRAS+ TP53+	**115**	**49**	**32.42 (23.05 - 78.61)**

Figure S2. Analysis of *KRAS*-mutant NSCLC patients co-mutated with TP53, related to Figure 1
Kaplan-Meier analysis of overall survival in *KRAS*-mutant NSCLC patients from TCGA (n = 340) based on the mutation status of *TP53*. We combined data from three studies, including Lung Adenocarcinoma (TCGA, Firehose Legacy), Lung Adenocarcinoma (TCGA, Nature 2014), and Lung Adenocarcinoma (TSP, Nature 2008), for a total of 1382 samples. Statistical significance was determined using Logrank test.

Figure S3

A

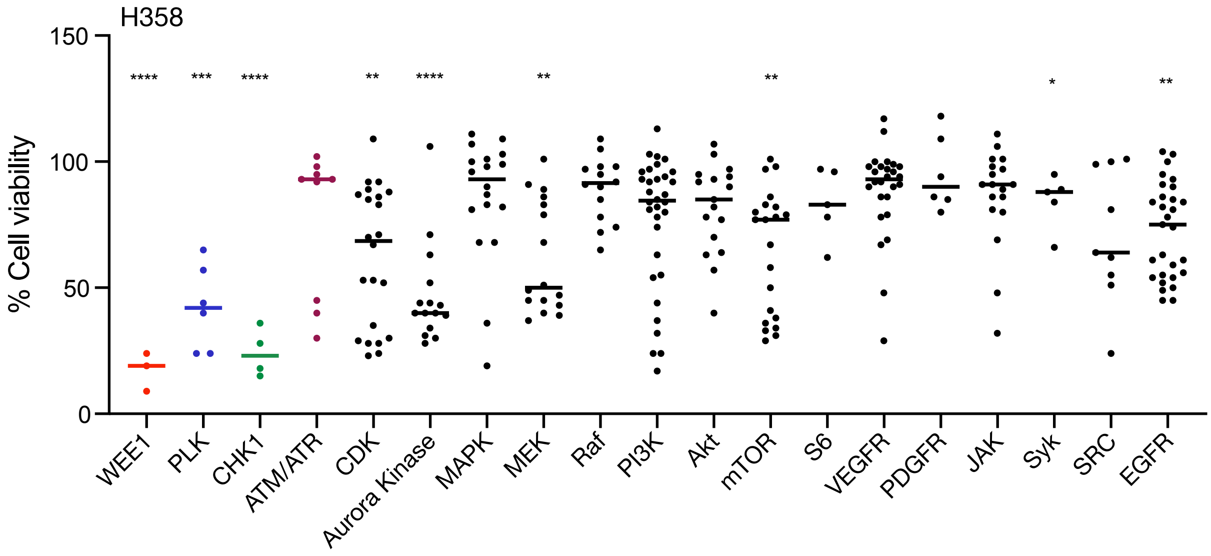

B

No	Agent	Target
1	PD016628	WEE1, CHK1
2	CUDC-907	HDAC, PI3K
3	CHIR-124	CHK1
4	Tivantinib (ARQ 197)	c-Met
5	GSK2126458 (GSK458)	PI3K, mTOR
6	AZD7762	CHK1
7	SC1	ERK
8	ZN-c3	WEE1
9	JNK Inhibitor IX	JNK
10	Flavopiridol HCl	CDK
11	AT7519	CDK
12	KX2-391	Src
13	HS-173	PI3K
14	Rigosertib (ON-01910)	PLK
15	HMN-214	PLK
16	Adavosertib	WEE1
17	BGT226 (NVP-BGT226)	PI3K, mTOR
18	BMS-754807	IGF-1R
19	PF-477736	CHK1, CHK2
20	Dinaciclib (SCH727965)	CDK
21	MK-8745	Aurora Kinase
22	SNS-032 (BMS-387032)	CDK
23	Flavopiridol (Alvocidib)	CDK
24	Lenvatinib (E7080)	VEGFR
25	Torin 2	mTOR

■ WEE1 ■ CHK1 ■ PLK ■ CDK

Figure S3. Drug screening of H358 cells, related to Figure 2

(A) H358 cells were treated with each compound from the library (1 μM). Cell viability was assessed by MTT assays at 72 hours. An overview of the growth inhibition of H358 by various pathway inhibitors is shown. (B) The top 25 agents that enhance growth inhibition of H358 are presented. Red clusters represent WEE1 inhibitors; green, CHK1 inhibitors; blue, PLK inhibitors; and gray, CDK inhibitors. Significant differences were determined by comparing the cells treated with DMSO using Student's *t*-test. Data are presented as mean ± SD of experimental replicates $*p < 0.05$, $**p < 0.01$, $***p < 0.001$, and $****p < 0.0001$.

Figure S4

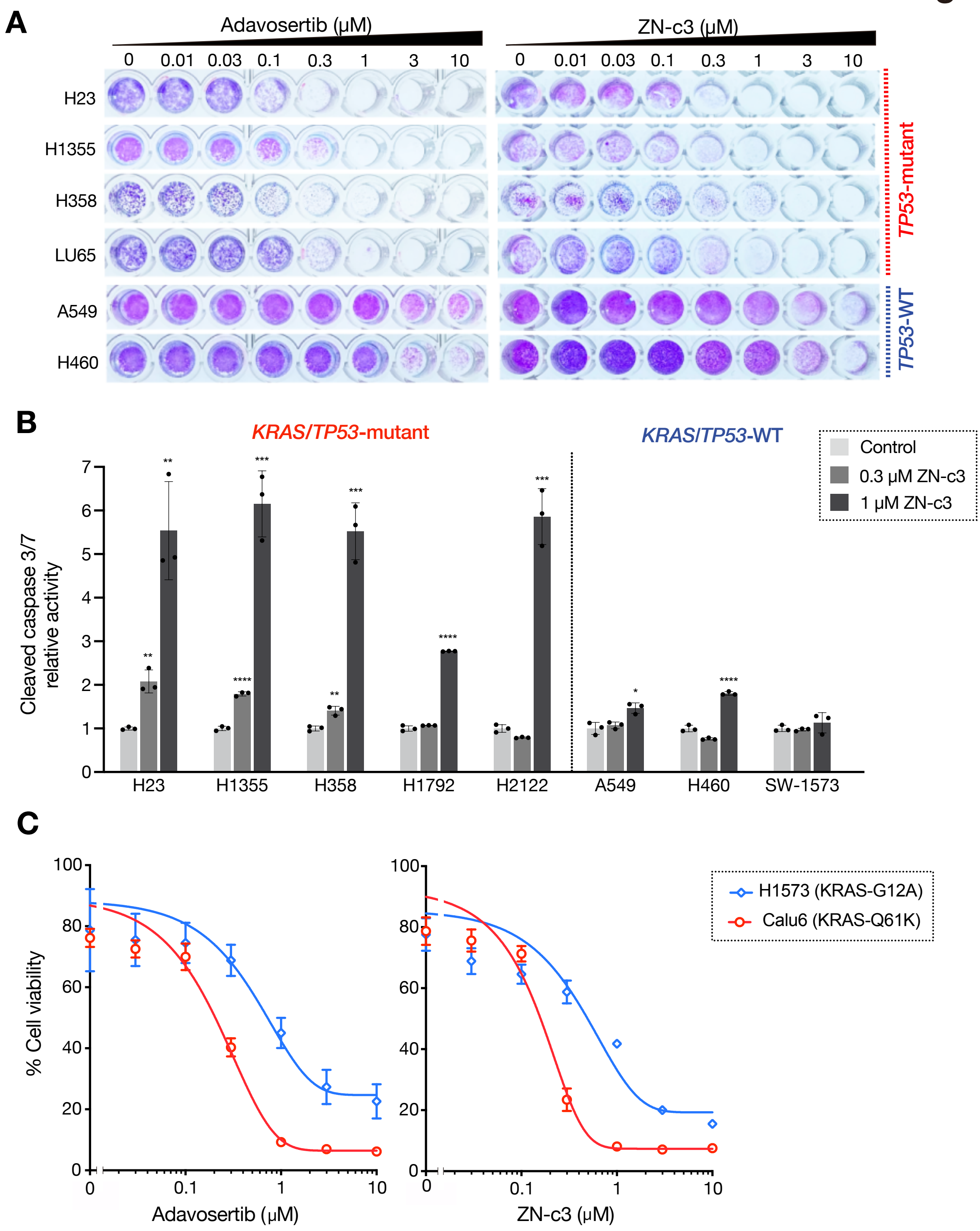

Figure S4. Effect of wee1 inhibitors on *KRAS*-mutant NSCLC cells, related to Figure 2
(A) H23, H1355, H358, LU65, A549, and H460 cells were treated with adavosertib or ZN-c3 at the indicated concentration. The cell growth was analyzed after 7 days using crystal violet staining. (B)H23, H1355, H358, H1792, H2122, A549, H460, and SW-1573 cells were treated with ZN-c3 at the indicated concentration for 48 hours. Apoptosis was quantified using the Caspase-Glo® 3/7 Assay. Bars represent mean ± SD of triplicate. Statistical significance was determined using Student' s t test. *$p < 0.05$, **$p < 0.01$, ***$p < 0.001$, and ****$p < 0.0001$. (C) H1573 and Calu6 cells were treated with adavosertib or volasertib at the indicated concentration. IC50 was assessed by MTT assay at 72 hours. Bars represent mean ± SD of triplicate.

Figure S5

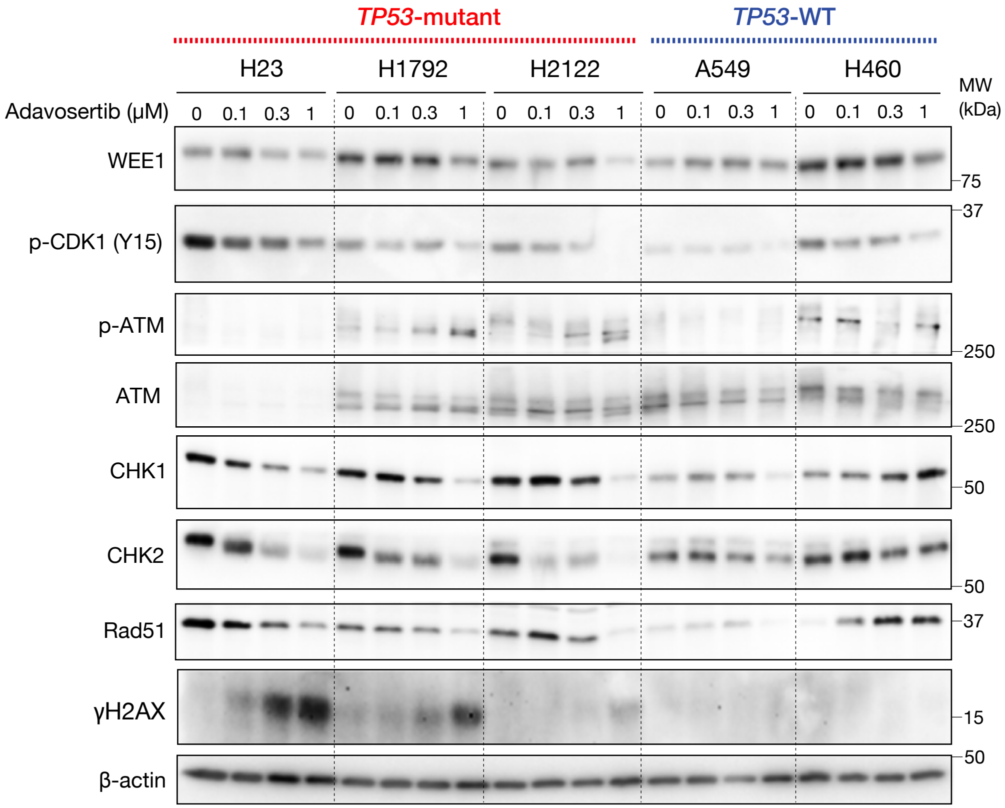

Figure S5. Effect of adavosertib on DDR pathways, related to Figure 4
H23, H1792, H2122, A549, and H460 were treated with adavosertib at the indicated concentration for 48 hours. Cell lysates were analyzed by western blotting with the indicated antibodies.

Figure S6

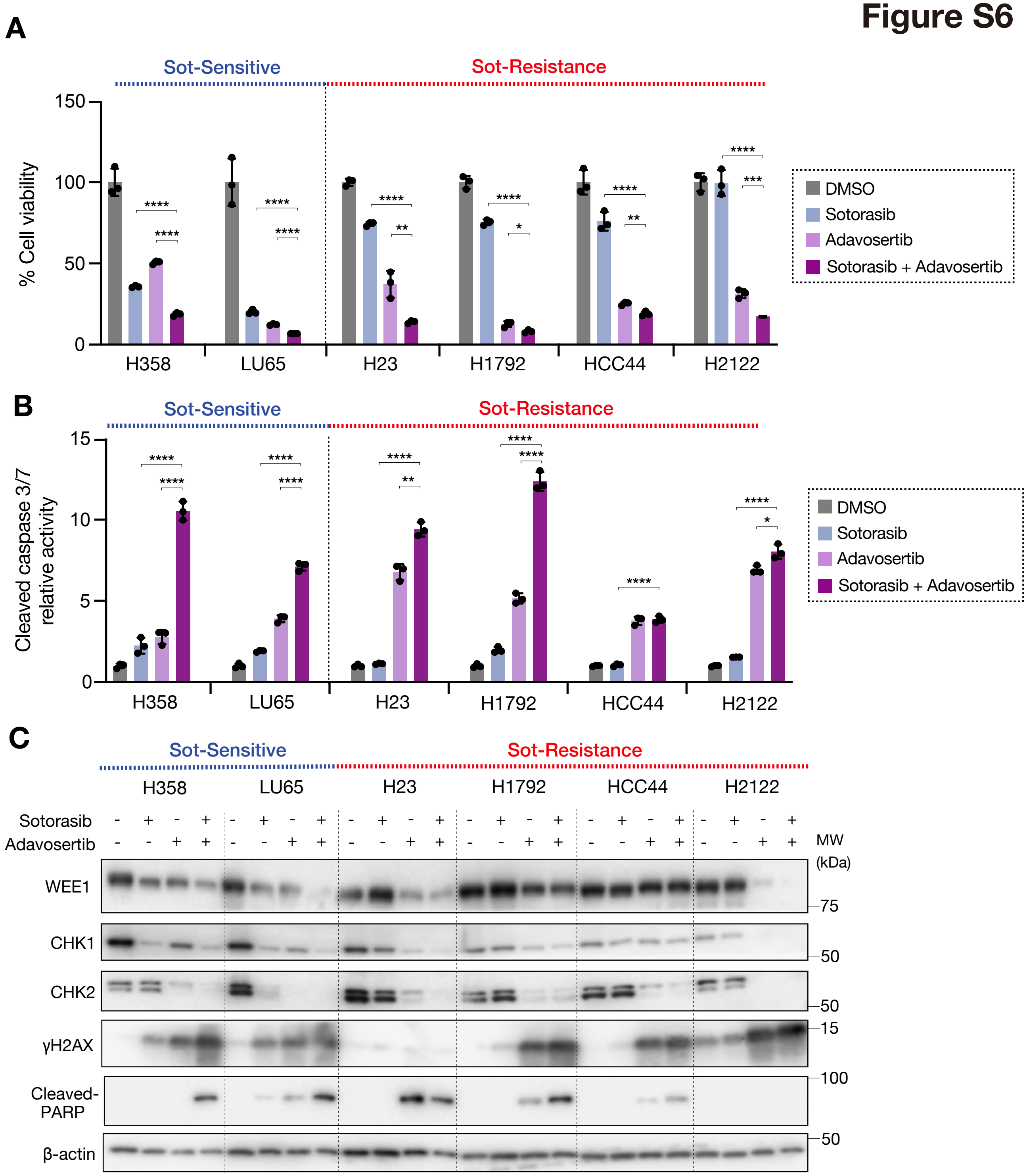

Figure S6. Effect of sotorasib in combination with adavosertib, related to Figure 5

(A) H358, LU65, H23, H1792, HCC44 and H2122 were treated with 1 μM Sotorasib and/or 1 μM adavosertib. The cell viability was assessed by MTT assay at 72 hours.Bars represent mean ± SD of triplicate. Statistical significance was determined using Student' s t test. *p < 0.05, **p < 0.01, ***p < 0.001, and ****p < 0.0001. (B) Apoptosis was quantified using the Caspase-Glo® 3/7 Assay at 48 hours. Bars represent mean ± SD of triplicate. Statistical significance was determined using Student' s t test. *p < 0.05, **p < 0.01, ***p < 0.001, and ****p < 0.0001.(C) Cell lysates were extracted at 48 hours and analyzed by western blotting with the indicated antibodies. Significant differences were determined using Student's *t*-test. Data are presented as mean ± SD of experimental replicates; *n* = 3, **p* < 0.05, ***p* < 0.01, ****p* < 0.001, and *****p* < 0.0001.

Figure S7

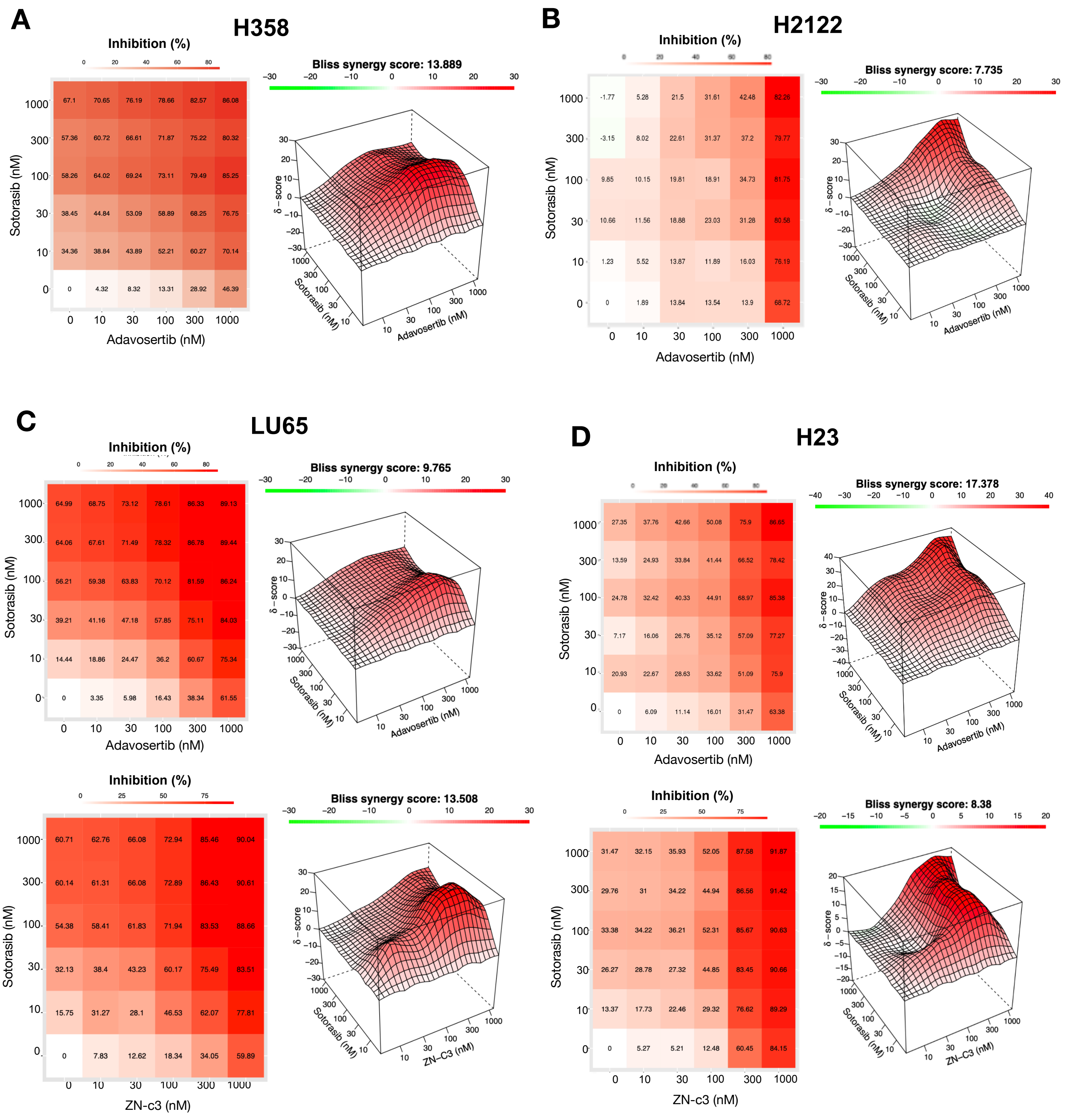

Figure S7. Synergistic effects of sotorasib and WEE1 inhibitor, related to Figure 5

(A) H358 cells were treated with adavosertib and sotorasib for 72 hours at the indicated concentration. Cell viability was assessed by MTT assay. 2-D surface response for cell inhibition and 3-D surface Bliss synergy response score were shown. (B) H2122 cells were treated with adavosertib and sotorasib for 72 hours at the indicated concentration. Cell viability was assessed by MTT assay. 2-D surface response for cell inhibition and 3-D surface Bliss synergy response score were shown. (C) LU65 cells were treated with adavosertib or ZN-c3 and sotorasib for 72 hours at the indicated concentration. Cell viability was assessed by MTT assay. 2-D surface response for cell inhibition and 3-D surface Bliss synergy response score were shown. (D) H23 cells were treated with adavosertib or ZN-c3 and sotorasib for 72 hours at the indicated concentration. Cell viability was assessed by MTT assay. 2-D surface response for cell inhibition and 3-D surface Bliss synergy response score were shown. All data are presented as mean of triplicates.

Figure S8

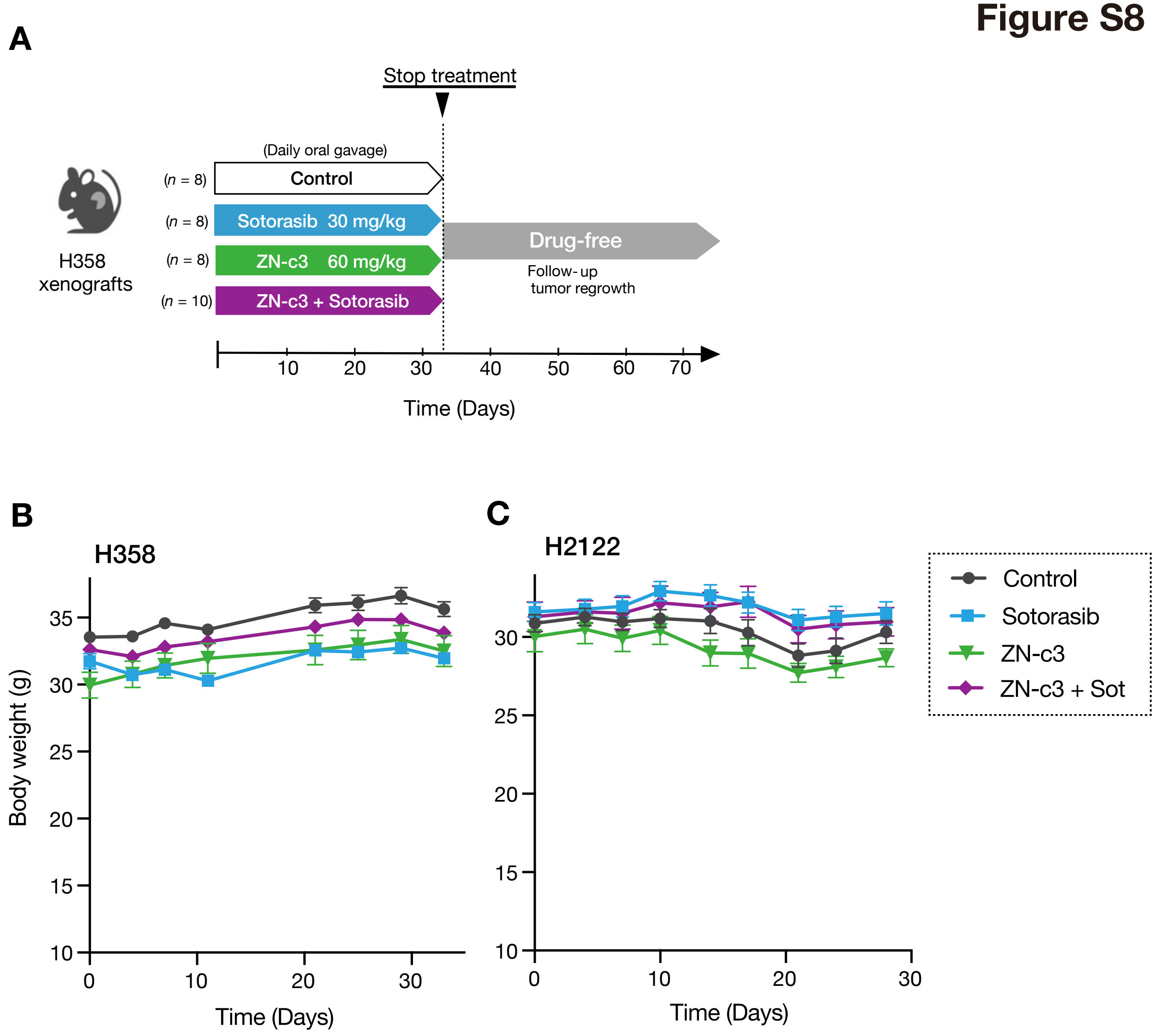

Figure S8. Additional *in vivo* experimental data, related to Figure 7

(A) Schematic of the in vivo experimental protocol of H358 xenograft model. (B) Percentage body weight changes in mice of H358 treated with vehicle (control: $n = 8$), sotorasib (30 mg/kg: $n = 8$), ZN-c3 (60 mg/kg: $n = 8$), or the combination of ZN-c3 (60 mg/kg) and sotorasib (30 mg/kg) ($n = 10$). (C) Percentage body weight changes in mice bearing H2122 xenografts treated with vehicle (control: $n = 8$), sotorasib (30 mg/kg: $n = 8$), ZN-c3 (60 mg/kg: $n = 8$), or the combination of ZN-c3 (60 mg/kg) and sotorasib (30 mg/kg) ($n = 10$). Bars represent mean ± SD.

Figure S9

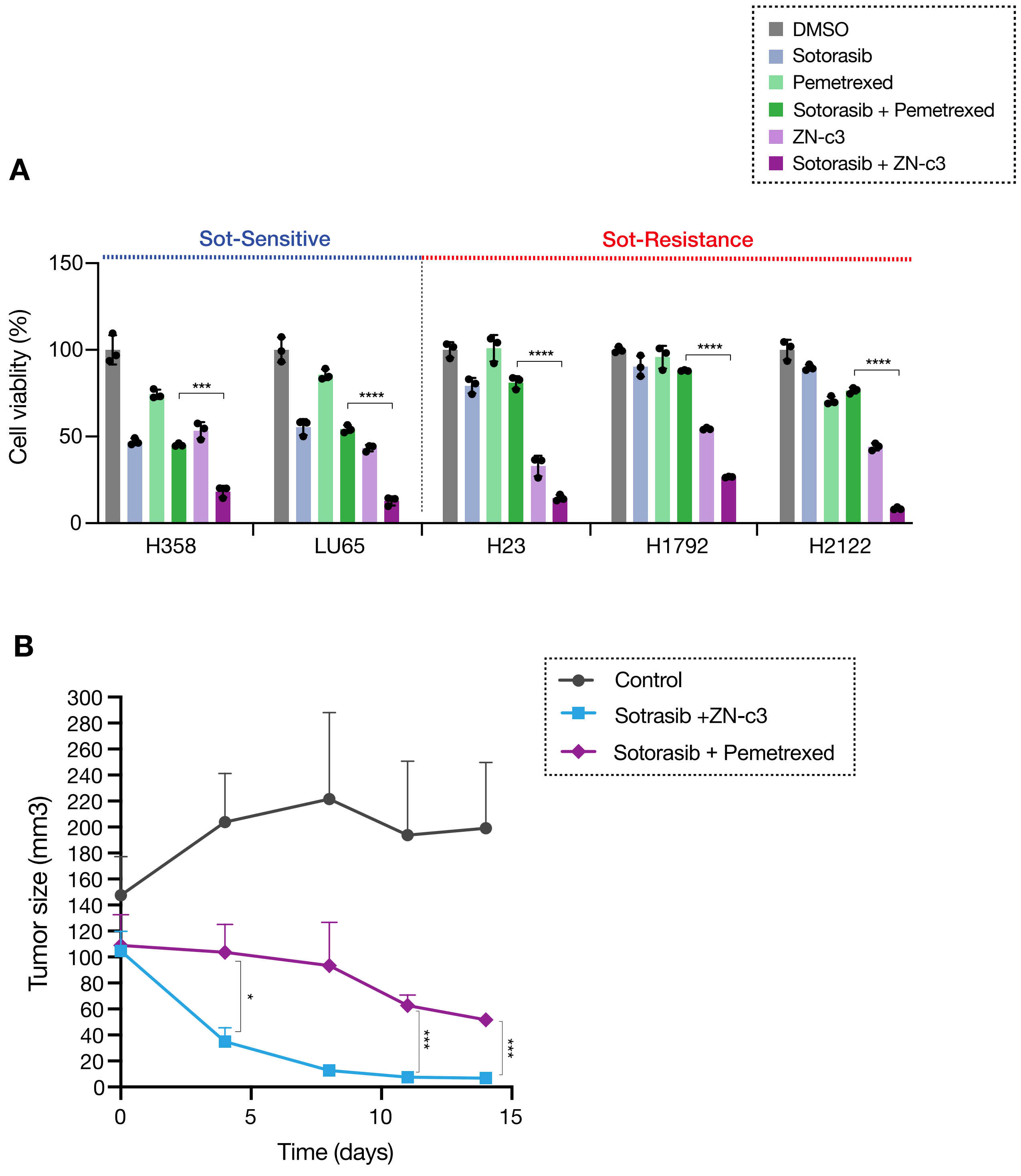

Figure S9. Effect of sotorasib in combination with pemetrexed, related to Figures 5 and 7.
(A) H358, LU65, H23, H1792, and H2122 were treated with 1 µM Sotorasib and/or 1 µM ZN-c3 and/or 100 nM Pemetrexed. The cell viability was assessed by MTT assay at 72 h. (B) Tumor volumes in mice bearing H358 xenografts treated with vehicle (control), the combination of sotorasib (30 mg/kg) and ZN-c3 (30 mg/kg), and the combination of sotorasib (30 mg/kg) and pemetrexed (50 mg/kg). Data are presented as mean ± SEM of experimental replicates; *n* = 3 Statistical significance was determined using Student' s t test.*p < 0.05 and ***p < 0.001.

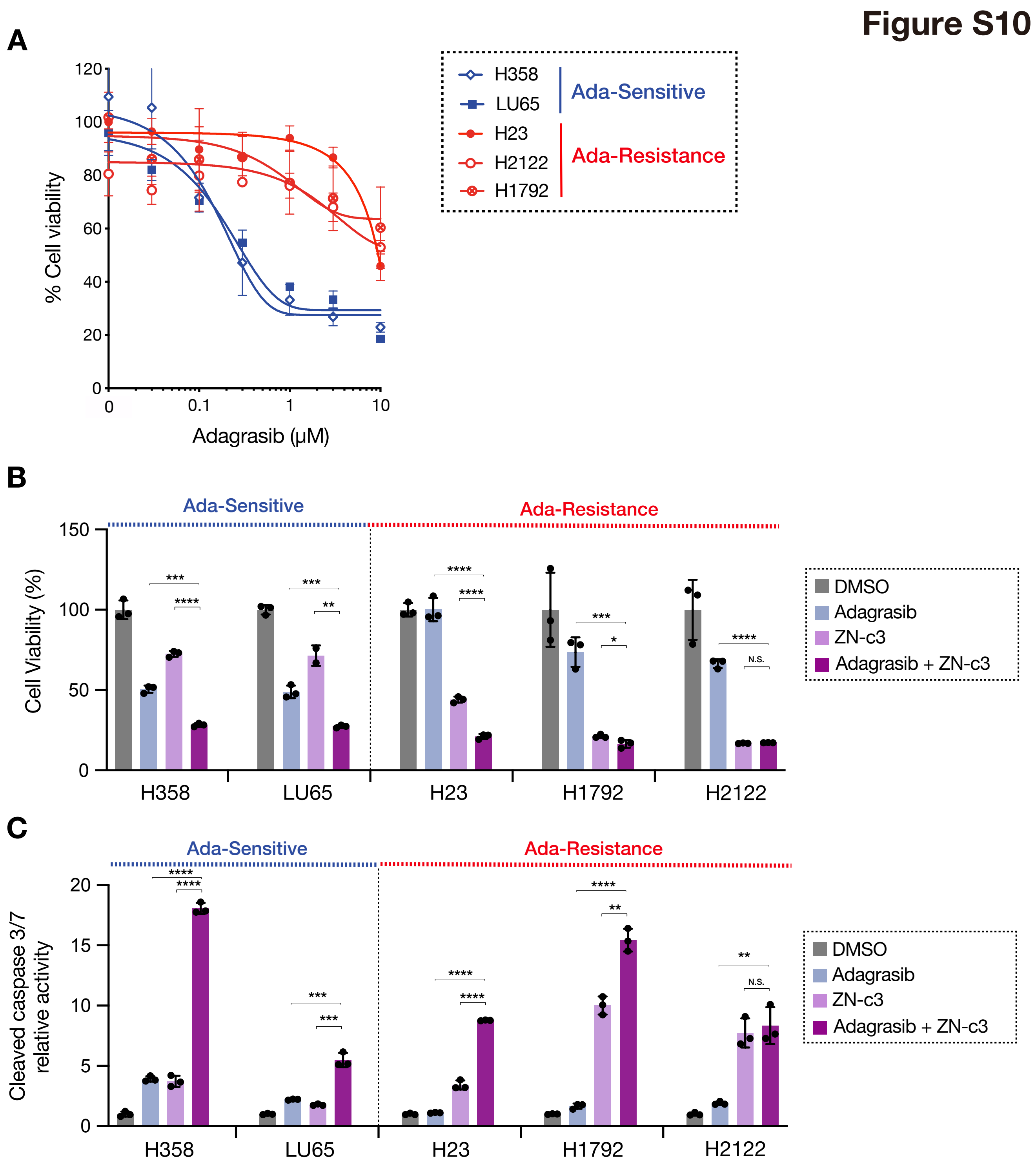

Figure S10. Effect of KRAS-G12C inhibitor, adagrasib, related to Figure 5.

(A)H358, LU65, H23, H1792, and H2122 cells were treated with adagrasib (Ada) for 72 h at the indicated concentration. The cell viability was assessed using a MTT assay. Bars represent mean ± SD of triplicate. (B) H358, LU65, H23, H1792, and H2122 cells were treated with 1 μM adagrasib and/or 1 μM ZN-c3. The cell viability was assessed using a MTT assay at 72 h. Bars represent mean ± SD of triplicate. Statistical significance was determined using Students t test. *p < 0.05, **p < 0.01, ***p < 0.001, and ****p < 0.0001. (C) Apoptosis was quantified using the Caspase-Glo® 3/7 Assay at 48 h. Bars represent mean ± SD of triplicate. Statistical significance was determined using Students t test. *p < 0.05, **p < 0.01, ***p < 0.001, and ****p < 0.0001.

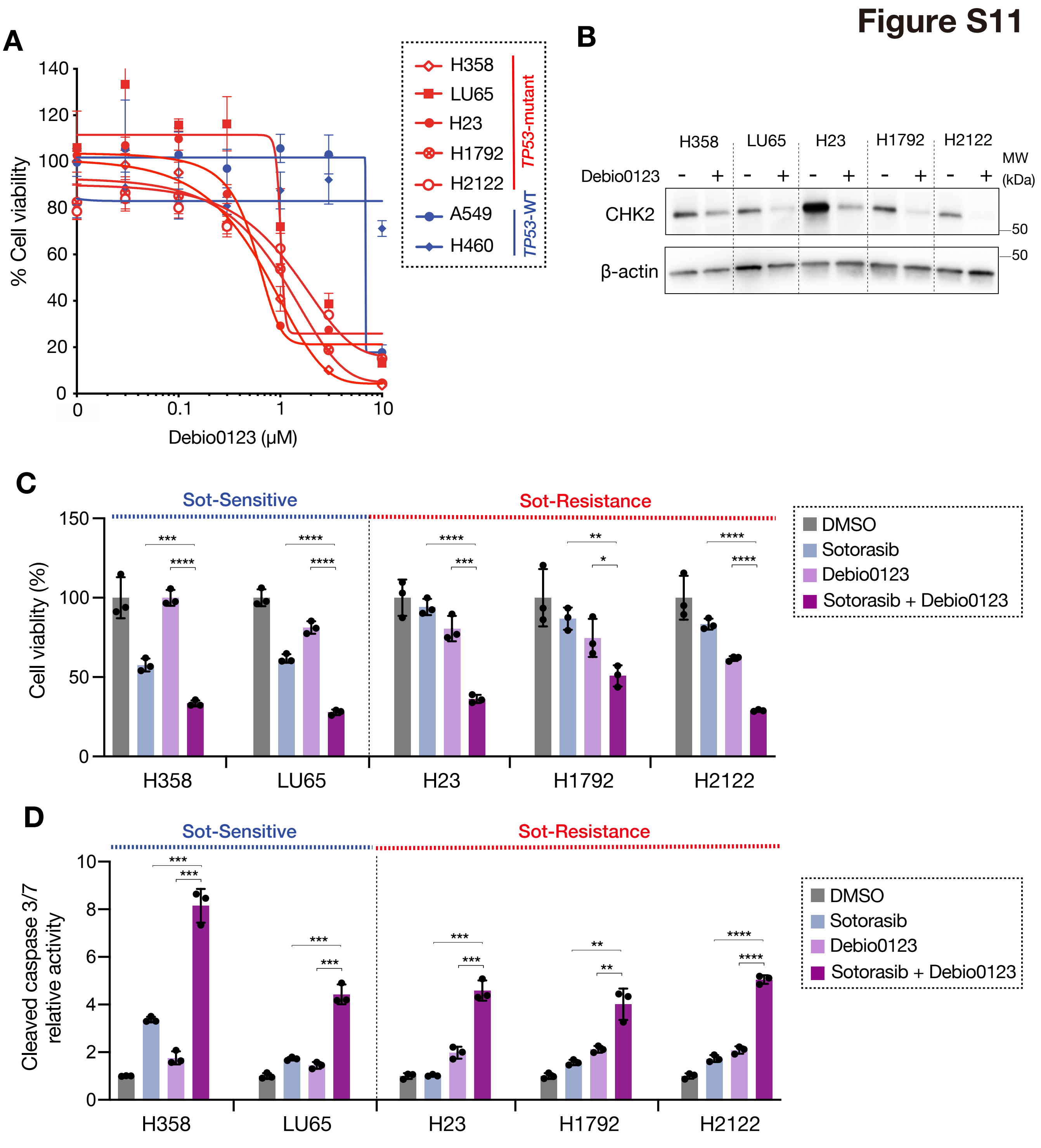

Figure S11. Effect of a next generation WEE1 inhibitor, Debio0123, related to Figure 5.
(A)H358, LU65, H23, H1792, H2122, A549, and H460 cells were treated with Debio0123 for 72 h at the indicated concentration. The cell viability was assessed using a MTT assay. Bars represent mean ± SD of triplicate. (B) Cell lysates were extracted at 48 h treatment of Debio0123 and analyzed by western blotting with the indicated antibodies. (C) H358, LU65, H23, H1792, and H2122 cells were treated with 1 μM sotorasib and/or 1 μM Debio0123. The cell viability was assessed using a MTT assay at 72 h. Bars represent mean ± SD of triplicate. Statistical significance was determined using Students t test. *p < 0.05, **p < 0.01, ***p < 0.001, and ****p < 0.0001. (D) Apoptosis was quantified using the Caspase-Glo® 3/7 Assay at 48 h. Bars represent mean ± SD of triplicate. Statistical significance was determined using Students t test. *p < 0.05, **p < 0.01, ***p < 0.001, and ****p < 0.0001.

Figure S12

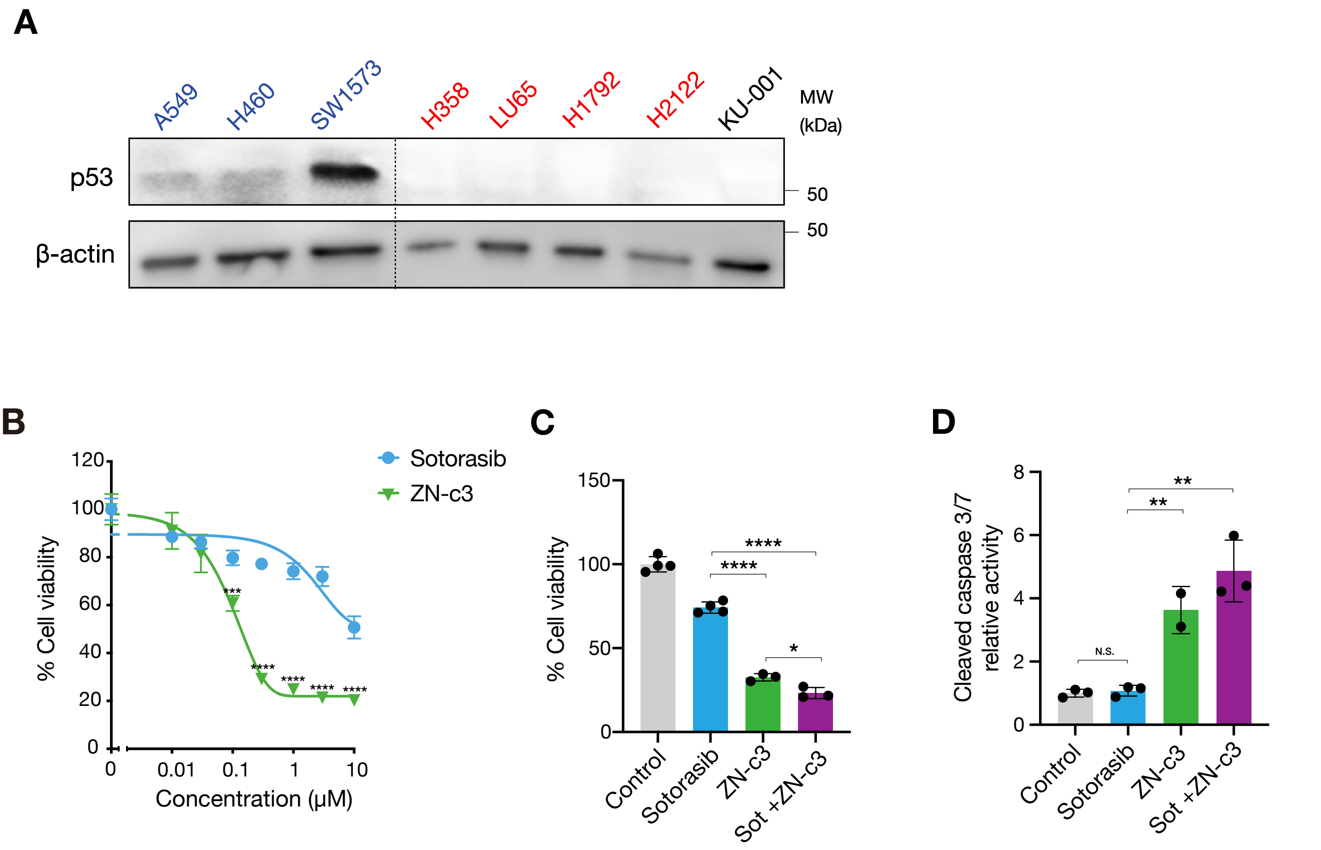

Figure S12. Additional KU-001 experimental data, related to Figure 7

(A)A549, H460, SW1573 (*TP53*-wild-type; blue), H358, LU65, H1792, and H2122 (*TP53*-mutant; red) and our established cell line KU-001 were analyzed by western blotting with the indicated antibodies.(B) KU-001 cell lines were treated with indicated concentrations of sotorasib or ZN-c3. Bars represent mean ± SD of triplicate. Statistical significance was determined using Student' s t test. ***$p < 0.001$, and ****$p < 0.0001$. (C) KU-001 cell lines were treated with 1 μM Sotorasib and/or 1 μM ZN-c3. The cell viability was assessed using MTT assay at 72 h. Bars represent mean ± SD of quadruplicate in control and Sotorasib) or triplicate in ZN-c3 and ZN-c3 + Sotrasib. Statistical significance was determined using Student' s t test. *$p < 0.05$ and ****$p < 0.0001$. (D) Apoptosis was quantified using the Caspase-Glo® 3/7 Assay at 48 h. p53 expression in cell lysates from *KRAS*-mutant NSCLC cell lines. Bars represent mean ± SD of triplicate. Statistical significance was determined using Students t test. **$p < 0.01$ and N.S.= non siginificance.

Figure S13

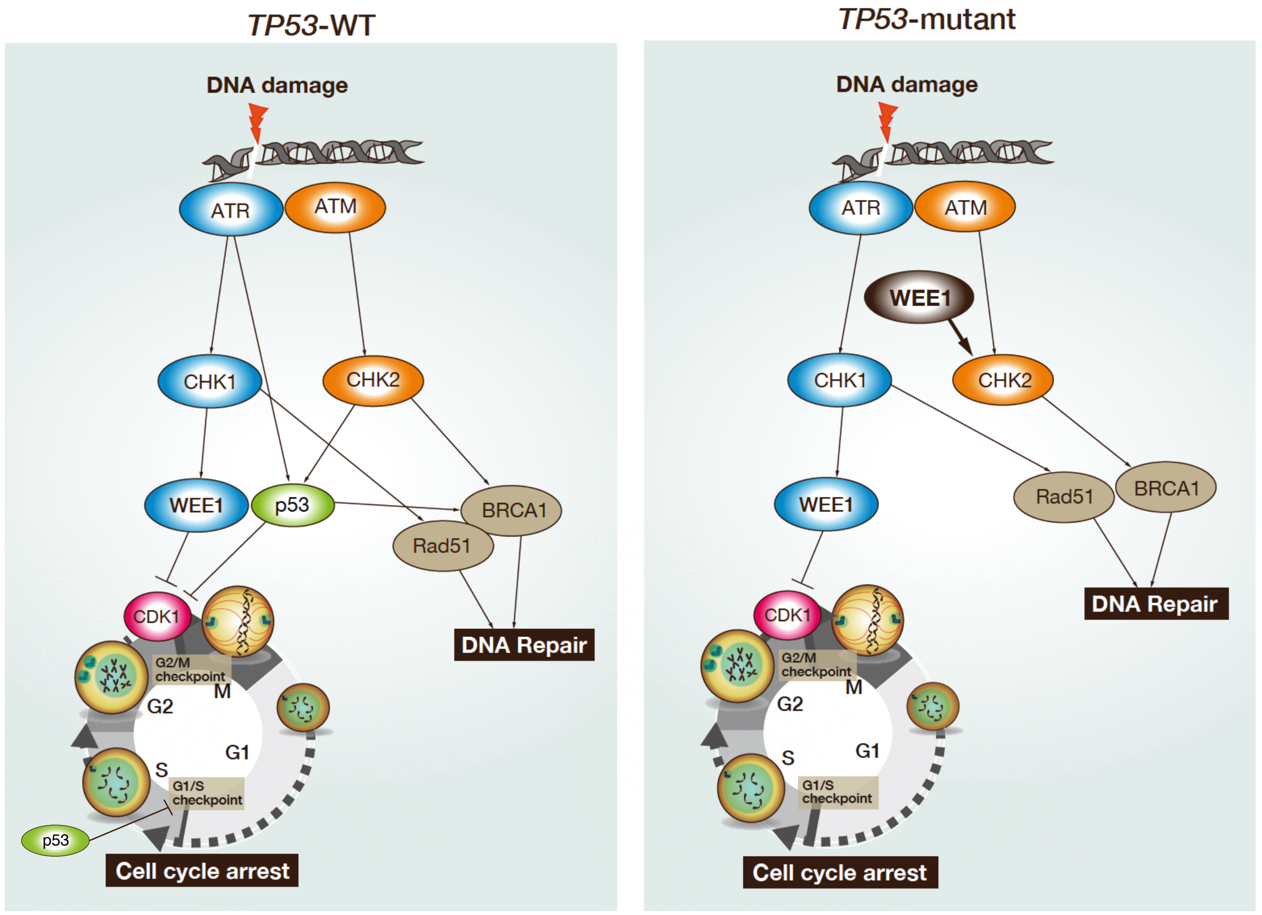

Figure S13. Role of WEE1 in DDR pathway, related to Figure 6.
Schematic of the hypothetical roles of WEE1 in TP53-WT or TP53-mutated KRAS-mutated NSCLC cells.

Figure S14

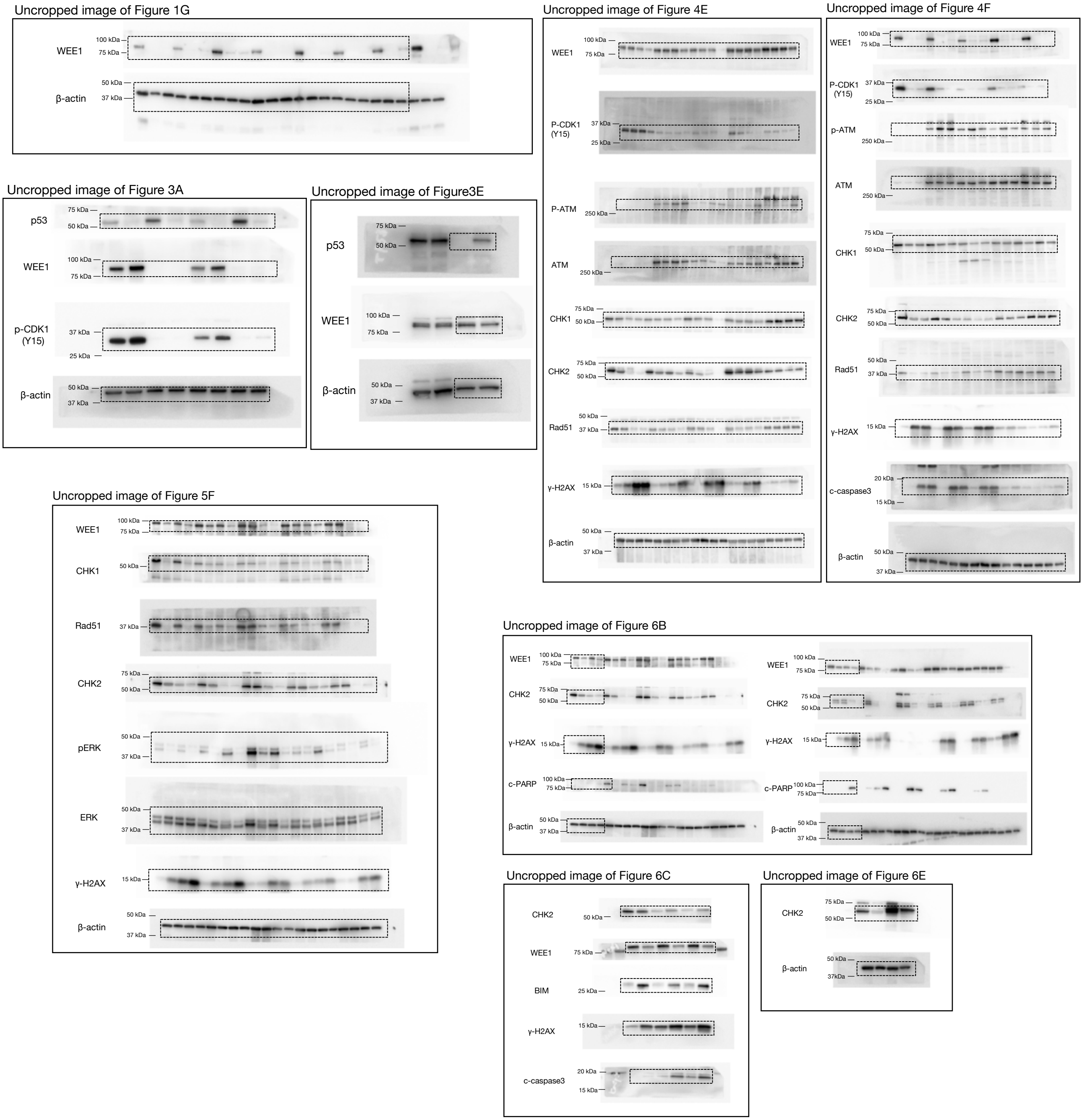

Figure S14. Uncropped image of Western blot analysis, related to Figures 1, 3, 4, 5 and 6
It provides the full, uncropped blot images to allow for the assessment of loading consistency and integrity across all samples related to main figures.

Table S1

Table S1: Summary of *KRAS* and *TP53* mutations in the cell lines, related to Figures 1, 2, and 5.

Cell lines	Aberration	*KRAS*	*TP53*
NCI-H23	H23	G12C-hetero	M246I (c.738G>C) -homo
NCI-H358	H358	G12C-hetero	delition -homo
NCI-H1355	H1355	G13C-hetero	Glu285Lys -homo
NCI-H1792	H1792	G12C-hetero	672+1G>A Splice donor site -homo
Lu-65	LU65	G12C-hetero	Glu11Gln -homo
NCI-H2122	H2122	G12C-homo	Q16L, C176F -hetero
NCI-H1573	H1573	G12A-hetero	R248L -homo
Calu-6	Calu6	Q61K-hetero	R196Ter -homo
HCC44	HCC44	G12C-homo	S94Ter, R175L -homo
A-549	A549	G12S-homo	WT
NCI-H460	H460	Q61H-homo	WT
SW1573	SW1573	G12C-homo	WT
